# Exploring the shared molecular mechanisms between systemic lupus erythematosus and primary Sjögren’s syndrome based on integrated bioinformatics and single-cell RNA-seq analysis

**DOI:** 10.3389/fimmu.2023.1212330

**Published:** 2023-08-08

**Authors:** Yanling Cui, Huina Zhang, Zhen Wang, Bangdong Gong, Hisham Al-Ward, Yaxuan Deng, Orion Fan, Junbang Wang, Wenmin Zhu, Yi Eve Sun

**Affiliations:** ^1^ Stem Cell Translational Research Center, Tongji Hospital, School of Medicine, Tongji University, Shanghai, China; ^2^ Shanghai Institute of Stem Cell Research and Clinical Translation, Shanghai East Hospital, School of Medicine, Tongji University, Shanghai, China; ^3^ Division of Rheumatology, Tongji Hospital of Tongji University School of Medicine, Shanghai, China

**Keywords:** systemic lupus erythematosus, primary Sjögren’s syndrome, bioinformatics, hub genes, TFs, scRNA-seq

## Abstract

**Background:**

Systemic lupus erythematosus (SLE) and primary Sjögren’s syndrome (pSS) are common systemic autoimmune diseases that share a wide range of clinical manifestations and serological features. This study investigates genes, signaling pathways, and transcription factors (TFs) shared between SLE and pSS.

**Methods:**

Gene expression profiles of SLE and pSS were obtained from the Gene Expression Omnibus (GEO). Weighted gene co-expression network analysis (WGCNA) and differentially expressed gene (DEG) analysis were conducted to identify shared genes related to SLE and pSS. Overlapping genes were then subject to Gene Ontology (GO) and protein-protein interaction (PPI) network analyses. Cytoscape plugins cytoHubba and iRegulon were subsequently used to screen shared hub genes and predict TFs. In addition, gene set variation analysis (GSVA) and CIBERSORTx were used to calculate the correlations between hub genes and immune cells as well as related pathways. To confirm these results, hub genes and TFs were verified in microarray and single-cell RNA sequencing (scRNA-seq) datasets.

**Results:**

Following WGCNA and limma analysis, 152 shared genes were identified. These genes were involved in interferon (IFN) response and cytokine-mediated signaling pathway. Moreover, we screened six shared genes, namely *IFI44L, ISG15, IFIT1, USP18, RSAD2* and *ITGB2*, out of which three genes, namely *IFI44L, ISG15* and *ITGB2* were found to be highly expressed in both microarray and scRNA-seq datasets. IFN response and ITGB2 signaling pathway were identified as potentially relevant pathways. In addition, STAT1 and IRF7 were identified as common TFs in both diseases.

**Conclusion:**

This study revealed *IFI44L, ISG15* and *ITGB2* as the shared genes and identified STAT1 and IRF7 as the common TFs of SLE and pSS. Notably, the IFN response and ITGB2 signaling pathway played vital roles in both diseases. Our study revealed common pathogenetic characteristics of SLE and pSS. The particular roles of these pivotal genes and mutually overlapping pathways may provide a basis for further mechanistic research.

## Introduction

Systemic lupus erythematosus (SLE) and primary Sjögren’s syndrome (pSS) are among the most common systemic autoimmune diseases and exhibit numerous shared clinical symptoms, serological profiles and immunological characteristics (1–3). Both SLE and pSS exhibit a predominance in females, and cases frequently present overlapping clinical symptoms, such as arthralgia, myalgia, and leukopenia (4). SLE and pSS preferentially target specific organs. SLE is characterized by a variety of disease-specific clinical manifestations, including skin rash, arthritis, lupus nephritis and hematological symptoms (5, 6). The pSS is a chronic inflammation condition that primarily affects the exocrine glands (salivary and lacrimal glands), resulting in oral and ocular dryness (7). Beyond affecting organs, peripheral blood plays an indispensable role in manifesting the immune pathophysiology for SLE and pSS. Peripheral blood mononuclear cells (PBMCs) are the immune cells most responsible for initiating the autoimmune inflammatory process against the target organs (8). Thus, the transcriptomic profiles of PBMC could provide pertinent insights into the molecular characteristics of the immune cells in SLE and pSS.

The etiologies and pathogeneses of SLE and pSS remain elusive and may be related to various factors, such as genetic predisposition, environmental triggers and epigenetic mechanisms (9). Genetic risk loci, including HLA class II, *IL12A* and *BLK* (associated with adaptive immunity), *IRF5* and *STAT4* (associated with innate immunity) are shared in these two diseases (10–12). Environmental factors such as Epstein-Barr virus (EBV) infection and alterations in gut microbial composition have been frequently observed in individuals with SLE and pSS (13–16). Various studies have reported that viral infections promote the development and progression of pSS through type I interferon (IFN). It has been demonstrated that the gene regulation by type I IFN is linked to an escalation disease activity in both SLE and pSS (9, 17–19). In recent studies, widespread changes in DNA methylation have been identified in SLE and pSS by epigenome-wide association studies (EWAS) (1, 20–22). Although these findings suggest the presence of common pathogenetic mechanisms between SLE and pSS, systematic cross-comparative analyses at the genetic level have yet to be conducted.

The rapid development of bioinformatics approaches has facilitated a more robust comprehension of disease pathobiology at the genetic level (23). The identification of common transcriptional features between SLE and pSS may provide valuable insights into shared pathogenetic characteristics of these two diseases. To this end, we performed comprehensive bioinformatics analyses in microarray and single-cell RNA sequencing (scRNA-seq) datasets to identify shared hub genes, related pathways and transcription factors (TFs) in SLE and pSS. We further investigated the correlation between hub gene and immune cell as well as related pathway, and validated their expression and location using scRNA-seq data. Finally, we predicted and verified TFs both in microarray and scRNA-seq datasets. Collectively, the shared hub genes, relevant pathways and TFs identified in this study have the potential to provide new insights to the genetic etiologies of SLE and pSS.

## Materials and methods

### Data source

Gene Expression Omnibus (GEO) (http://www.ncbi.nlm.nih.gov/geo/) is an extensive and publicly available database that contains numerous high-throughput sequencing and microarray datasets related to many diseases. The keywords “systemic lupus erythematosus” and “primary Sjögren’s syndrome” were used to search SLE and pSS gene expression datasets. The selected datasets for analysis strictly consisted of gene expression profiles for both cases and controls, generated from the same sequencing platform and exclusively from human specimens. Datasets GSE50772 (24), GSE81622 (25) and GSE135779 (26) were selected for SLE; GSE84844 (27), GSE48378 (28) and GSE157278 (29) were selected for pSS. The datasets were downloaded from GEO for subsequent analyses. For SLE, dataset GSE50772 includes 61 SLE samples and 20 healthy control samples (Platform: GPL570 Affymetrix Human Genome U133 Plus 2.0 Array); GSE81622 contains 30 SLE samples and 25 healthy control samples (Platform: GPL10558 Illumina HumanHT-12 V4.0 expression bead chip); and GSE135779 consists of 42 SLE samples and 17 control samples (Platform : GPL20301 Illumina HiSeq 4000). For pSS, dataset GSE84844 includes 30 pSS samples and 30 healthy control samples (Platform : GPL570); GSE48378 contains 11 pSS samples and 16 healthy control samples (Platform: GPL5175 Affymetrix Human Exon 1.0 ST Array); and GSE157278 consists of 5 pSS samples and 5 control samples (Platform: GPL24676 Illumina NovaSeq 6000). For microarray datasets, the series matrix files provided by the contributors include data processed by MAS5 or RMA algorithms. We read the data with the GEOquery package and matched the probes to their gene symbols according to the annotation documents of corresponding platforms. Finally, the gene matrix with row names as gene symbols and column names as sample names was obtained for subsequent analyses.

### Weighted gene co-expression network analysis

To identify gene co-expression modules associated with SLE and pSS, we conducted weighted gene co-expression network analysis (WGCNA) on the GSE50772 and GSE84844 datasets. The WGCNA R package was used to conduct the analysis (30). We selected the top 5000 genes of the median absolute deviation in the expression matrix of the dataset for WGCNA. Prior to the analysis, the ‘Hclust’ function was used to eliminate outlier samples. The parameters were networkType = “signed” and TOMType = “signed”. We then selected an optimal soft threshold ranging from 1 to 20 using the ‘pickSoftThreshold’ function to build an adjacency matrix, which was then transformed into a topological overlap matrix (TOM). Co-expression modules were identified through hierarchical clustering, followed by Pearson correlation analysis to compute the correlation between the module eigengene and clinical feature to obtain the expression profile of each module. We then chose the modules with high correlation coefficients with SLE and pSS and obtained genes from these modules for further analysis.

### Identification of DEGs

The differentially expressed genes (DEGs) in SLE and pSS were determined by using the limma R package (31). First, the GSE50772 and GSE84844 datasets were converted into an expression matrix and grouped. Next, the limma package was used to normalize and analyze the datasets to obtain DEGs. Genes with adjusted *p*-value [false discovery rate (FDR)] < 0.05 and |log2FC (fold change) | ≥ 0.5 were considered as DEGs (32). Furthermore, genes were classified as upregulated or downregulated based on their log2FC value being greater than 0.5 or less than -0.5, respectively. The overlapping DEGs of SLE and pSS were identified by using an online Venn diagram tool.

### Functional enrichment analysis

Gene ontology (GO) is a comprehensive resource regarding the functions of genes and gene products, providing annotations for gene products related to molecular functions, biological processes, and cellular components (33). Hallmark gene sets represent biological states or processes derived from the Molecular Signatures Database (MSigDB) (34). The “clusterProfiler” R package was used to conduct GO and Hallmark functional annotation analyses. Significantly enriched outcomes were recognized by *p*-values less than 0.05.

### PPI network construction and module analysis

STRING is an online search tool for the retrieval of interacting genes (STRING; http://string-db.org) (35). WGCNA results and DEGs were combined and imported into the STRING database to construct the protein- protein interaction (PPI) network; the interaction score used for the PPI network was set at > 0.4. Analysis of the PPI network and visualization were carried out using Cytoscape (http://www.cytoscape.org) (36). The molecular complex detection technology (MCODE), a Cytoscape plug-in, was used to conduct key functional module analysis. The employed parameters were as follows: degree cutoff = 2, max depth = 100, node score cutoff = 0.2 and K-core = 2.

### Selection and validation of hub genes

The 96 common module genes and 91 common DEGs were combined and subsequently imported into the STRING database to construct a PPI network. To identify hub genes, the cytoHubba plug-in Cytoscape was applied (37). Five algorithms (MCC [maximal clique centrality], MNC [maximum neighborhood component], Closeness, Radiality and EPC [edge percolated component]) were used from cytoHubba to identify and validate the hub genes.

In order to verify the hub genes expression, the GSE81622 and GSE48378 datasets were downloaded. The GSE81622 dataset includes PBMC expression data from 25 patients diagnosed with SLE and 30 healthy controls. The GSE48378 dataset contains expression data of 11 patients diagnosed with pSS and 16 healthy controls. The Shapiro-Wilks test was performed in R to test for the normality of the variables. The w-value was close to 1, and *p*-value > 0.05. The comparison was then performed using the t-test in these two datasets, separately (38); *p*-values < 0.05 were considered significant.

### Pathways analysis and the correlation with hub genes

Gene Set Variation Analysis (GSVA) is a non-parametric and unsupervised methodology that is employed to evaluate gene set enrichment (GSE) in gene expression microarray and RNA-seq data (39). The GSVA R package was used to find the related pathways in SLE and pSS, by quantifying the activities of the 50 hallmark pathways. Correlations between hub gene and pathway were evaluated by Pearson correlation coefficient. R packages “ggplot2” and “pheatmap” were used for visualization.

### Single-cell RNA-Seq data analysis

The scRNA-seq datasets GSE135779 for SLE and GSE157278 for pSS were downloaded from GEO. Down-stream analyses were performed using the Seurat R package (version 4.1.0) (40). Following quality control (QC), cells with fewer than 200 expressed genes and >10% mitochondria-related genes were excluded. After normalization, the top 3000 highly variable genes (HVGs) in each Seurat object were selected for subsequent analysis, including ScaleData, RunPCA, RunTSNE and RunUMAP. The cells were then clustered using the FindNeighbors and FindClusters functions. The generated clusters were visualized using uniform manifold approximation and projection (UMAP) plot. Cell types were identified using classical marker genes and the SingleR algorithm (41). The gene list used to generate IFN-response score comprises the following *IFI27, IFI6, RSAD2, IFI44, IFI44L, IFITM1, IFNGR1, IFIT2, MX2, OASL, GBP1, USP18, LY6E, OAS1, SIGLEC1, ISG15, IFIT1, OAS3, HERC5, MX1, LAMP3, EPSTI1, IFIT3, OAS2, RTP4, PLSCR1, DNAPTP6, TYK1* and *CXCL10* (42–45). The AddModuleScore function in Seurat R was used to calculate the IFN-response score. Differential expression analysis was performed on scRNA-seq datasets using the “FindMarkers” function in the Seurat package with default parameters. This analysis aimed to compare the expression profiles of different cell types between different groups (SLE/HC and pSS/HC). Adjusted *p*-value < 0.05 and |log2FC| > 0.25 was used to define significant DEGs.

### Estimation of immune cell fractions and the correlation with hub genes

CIBERSORTx is a suite of machine learning tools designed for detecting the abundance of cell types in bulk RNA-seq and microarray data (46, 47). We used the GSE135779 and GSE157278 scRNA-seq datasets to build scRNA-seq signature matrices with CIBERSORTx, respectively. After following the instruction to format and upload the single-cell reference matrix file, we ran the “Create Signature Matrix” module to build the scRNA-seq signature matrix (47). We used the generated signature matrices to perform CIBERSORTx deconvolution on the GSE50772 and GSE84844 datasets, separately. *p*-value < 0.05 was considered statistically significant. To visualize the proportion of each immune cell type, boxplots were constructed, with red and blue color-coding to indicate disease and healthy control (HC) status, respectively. The correlation between each hub gene and immune cell type was evaluated by Pearson correlation coefficient. R packages “ggplot2” and “pheatmap” were used for visualization.

### Cell-cell communication analysis

The CellChat package is a powerful tool that facilitates the quantitative inference and analysis of intercellular communication networks from scRNA-seq data. CellChat is capable of predicting the major signaling inputs and outputs for cells, as well as how these signals coordinate for various cellular functions. Once cell types have been identified, CellChat can be further used to analyze cell-cell communication (48). In this study, we generated new CellChat objects from the Seurat objects. The CellChatDB was set as the reference database. The two scRNA-seq data were further divided into two groups each based on their respective conditions (HC vs SLE, HC vs pSS). Thereafter, the variations in ligand-receptor interactions and signaling pathways among these states were thoroughly examined.

### Prediction and verification of transcription factors

iRegulon is a computational method to reverse-engineer the transcriptional regulatory network underlying a co-expressed gene set using cis-regulatory sequence analysis. This method utilizes a genome-wide ranking-and-recovery approach to detect enriched transcription factor (TF) motifs and their optimal sets of direct targets (49). In this study, we employed iRegulon to predict the TFs of hub genes, and their expression levels were subsequently validated in microarray datasets. Furthermore, we confirmed the expression and localization of these TFs in scRNA-seq data. The major parameters in iRegulon were the following: Species and gene nomenclature = “Homo sapiens, HGNC symbols”, Motif collection = “10K (9713 PWMs)”, Track collection = “1120 ChIP-seq tracks (ENCODE raw signals)”, Putative regulatory region = “20kb centered around TSS”, Enrichment score threshold = 3.0, ROC threshold for AUC calculation = 0.03 and the rank threshold = 5000.

### Gene regulatory network

Single-cell regulatory network inference and clustering (SCENIC) is a computational method to infer cell type-specific gene regulatory networks (GRNs) from scRNA-seq data (50). The input matrices were the raw unique molecular identifier (UMI) counts for each sample obtained from Seurat. Genes present in RcisTarget’s databases (hg19-500 bp-upstream-7species. mc9nr. feather and hg19-tss-centered-10 kb-7species. mc9nr.feather) were utilized. Following the SCENIC pipeline, the GENIE3 method and GRNBoost were used to identify potential TF targets, and the regulon-specific score (RSS) was generated. Only significantly upregulated regulons were involved in further analysis.

## Results

### GEO information

**Figure 1:**
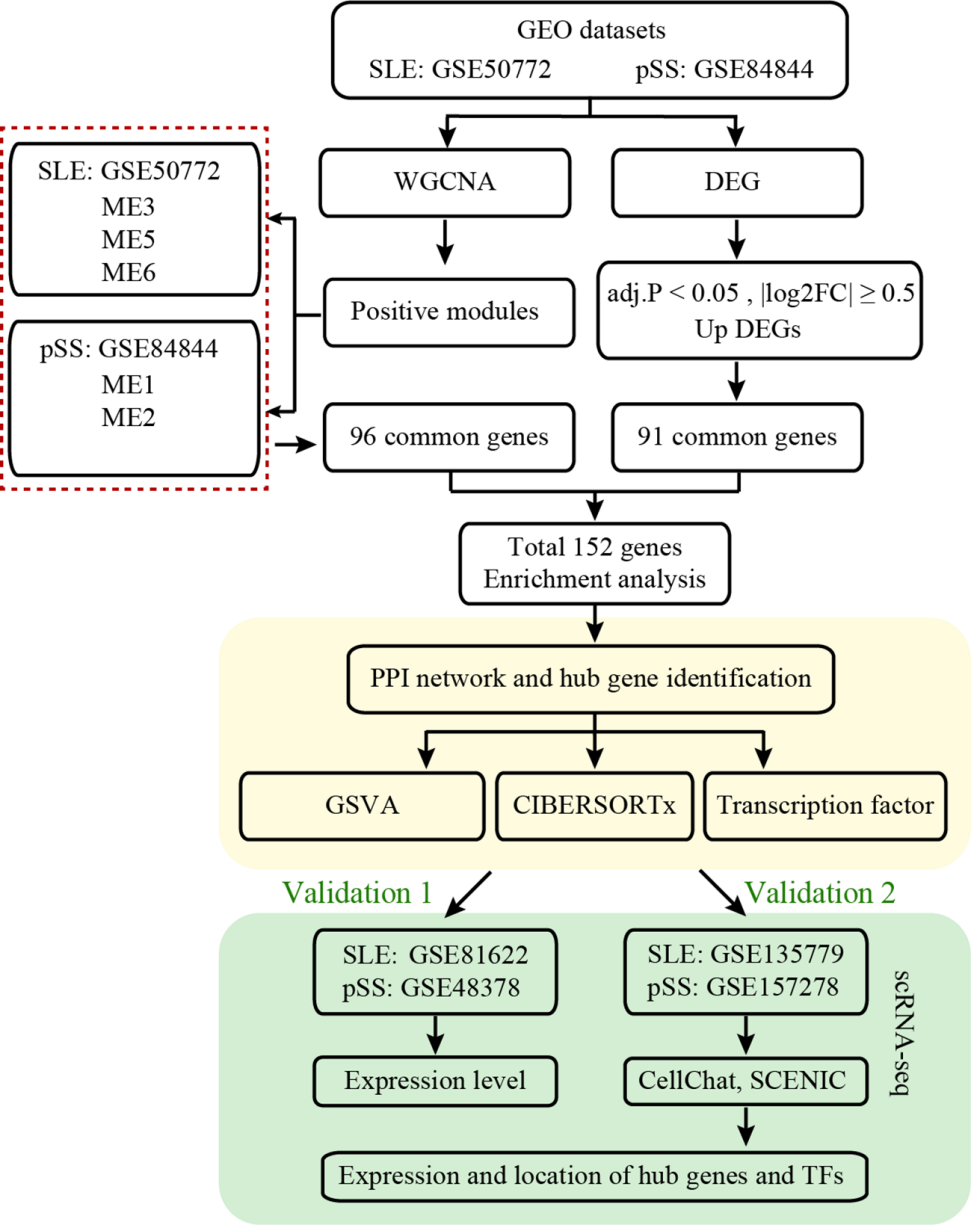
Workflow diagram of this work.

The workflow of this study is illustrated in **Figure 1**. Four microarray datasets, including GSE50772, GSE81622, GSE84844 and GSE48378, along with two scRNA-seq datasets, namely GSE157278 and GSE135779, were downloaded from GEO. Information from these datasets, including GSE number, detection platforms, samples and source types, is provided in **Supplementary Table 1**. WGCNA, DEGs, GSVA and immune cell analyses were performed on GSE50772 and GSE84844 datasets. Expression levels for hub genes and TFs were validated using GSE81622 and GSE48378. Additionally, the hub genes and TFs expression patterns were further validated in scRNA-seq datasets, namely GSE135779 and GSE157278.

### Weighted gene co-expression network analysis of SLE and pSS

**Figure 2:**
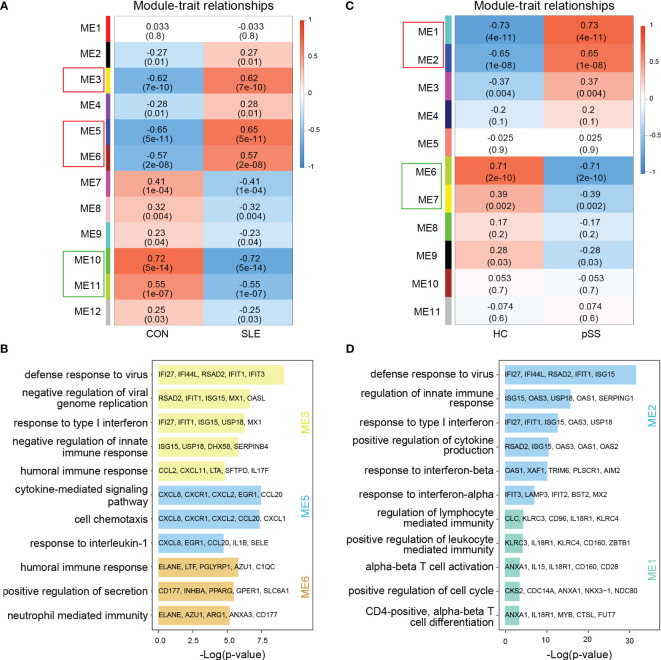
Weighted gene co-expression network analysis (WGCNA) and GO (Gene ontology) analysis of GSE50772 and GSE84844 datasets. **(A)** Heatmap of module-trait relationships in SLE. Each cell contains the corresponding correlation and *p*-value. **(B)** GO biological process analyses of three positively related modules with SLE. **(C)** Heatmap of module-trait relationships in pSS. Each cell contains the corresponding correlation and *p*-value. **(D)** GO biological process analyses of two positively related modules with pSS. SLE, systemic lupus erythematosus; pSS, primary Sjögren’s syndrome.

In WGCNA, the module-trait relationship heatmap according to the Pearson correlation coefficient showed the correlation between each module and the clinical trait. After processing with ‘Hclust’, one SLE sample was eliminated in GSE50772 dataset, and two pSS samples were eliminated in GSE84844 (**Supplementary Figure 1**). A total of 12 modules were identified in GSE50772, and 11 modules were identified in GSE84844. Afterwards, the correlation between each module and clinical trait was calculated. In GSE50772 database, the ME3, ME5 and ME6 modules had high positive correlations with SLE (r = 0.62, 0.65 and 0.57), comprising 1120 genes. The ME10 and ME11 modules were negatively correlated with SLE (r = -0.72 and -0.55), and comprised a total of 453 genes (**Figure 2A**). In GSE84844 database, the ME1 and ME2 modules showed high positive correlation with pSS (r = 0.73 and 0.65), containing 2796 genes. The ME6 and ME7 modules had negative correlations with pSS (r = -0.71 and -0.39), comprising a total of 637 genes (**Figure 2C**).

Further, we performed GO enrichment analysis on the positively related modules. For SLE, our results showed that the ME3 module was mainly associated with type I IFN response and innate immune response. The ME5 module was mainly related to cell chemotaxis and cytokine-mediated signaling pathway. Additionally, the ME6 module was involved in immune response (**Figure 2B**). For pSS, functional enrichment analysis indicated that the ME1 module was mainly associated with T cell activation and differentiation, and ME2 module was related to type I IFN response and cytokine production (**Figure 2D**). Therefore, type I IFN response and cytokine-mediated signaling pathway collectively participated in the pathogenesis of SLE and pSS.

### Enrichment analysis of common gene from WGCNA

**Figure 3:**
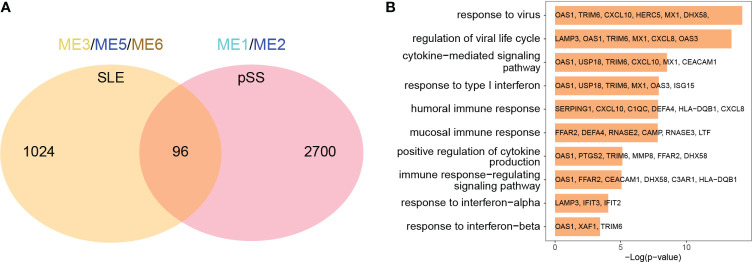
Venn diagrams and enrichment analysis of common genes from WGCNA. **(A)** Venn diagrams showing the overlap genes in positive related modules in SLE and pSS. **(B)** GO enrichment analysis of the 96 common genes. GO, gene ontology; SLE, systemic lupus erythematosus; pSS, primary Sjögren’s syndrome.

The common genes were screened between SLE positively related modules (ME3, ME5 and ME6 modules) and pSS positively related modules (ME1 and ME2 module). 96 genes overlapped in positively related modules from SLE and pSS (**Figure 3A**). Enrichment analysis results showed that the 96 genes were mainly associated with type I IFN response and cytokine-mediated signaling pathway (**Figure 3B**). There were 4 genes that overlapped in negatively related modules from SLE and pSS (**Supplementary Figures 2A, B**).

### Identification and function analyses of common DEGs

**Figure 4:**
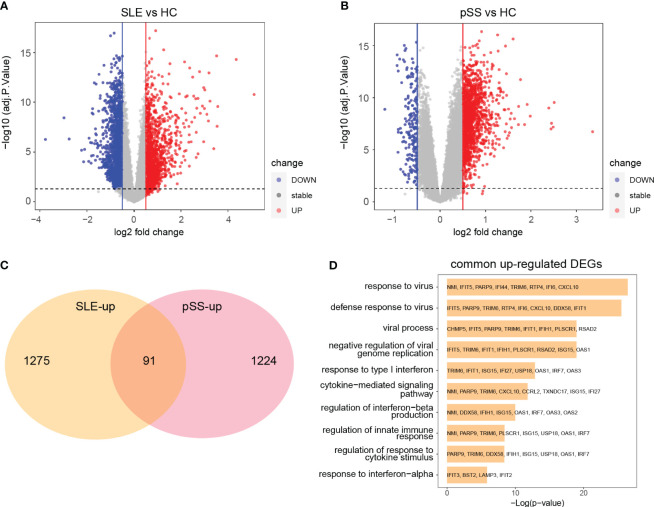
Identification common DEGs and functional enrichment analysis. **(A)** Volcano plot of GSE50772. **(B)** Volcano plot of GSE84844. Red dots indicate upregulated genes and blue dots indicate downregulated genes. **(C)** 91 upregulated DEGs overlapped in the two datasets. **(D)** GO enrichment analysis of common upregulated DEGs. DEGs, differentially expressed genes; GO, gene ontology; SLE, systemic lupus erythematosus; pSS, primary Sjögren’s syndrome.

The limma R package was utilized to perform an analysis of DEGs on the GSE50772 and GSE84844 datasets. Volcano plots showed the identified DEGs. For SLE dataset GSE50772, 2918 DEGs were identified, among which 1366 genes were upregulated and 1552 genes were downregulated (**Figure 4A**; **Supplementary File 1**). 1597 DEGs were obtained from the pSS dataset GSE84844, out of which 1315 DEGs were upregulated and 282 DEGs were downregulated (**Figure 4B**; **Supplementary File 2**). After examining the intersection for the DEGs, 91 shared upregulated DEGs and 11 shared downregulated DEGs were identified. The overlapping DEGs were visualized by Venn diagrams (**Figure 4C**; **Supplementary Figure 2C**, **Supplementary File 3**). To further analyze the underlying biological information associated with the common DEGs, GO analysis was performed. The results showed that the commonly upregulated DEGs were mainly enriched in type I IFN and cytokine stimulus response, which were consistent with the results of WGCNA (**Figure 4D**). These findings strongly indicated that type I IFN response and cytokine stimulus jointly participated in the development and progression of these two autoimmune diseases. We also performed the GO analysis on the upregulated DEGs in SLE and pSS, respectively. In addition to type I IFN response, inflammatory, immune response and T cell activation were also significantly enriched in SLE (**Supplementary Figure 3A**). For the upregulated DEGs in pSS, response to tumor necrosis factor (TNF), I-kappa B kinase/NF−kappa B signaling were also enriched (**Supplementary Figure 3B**).

### Selection and analysis of hub genes

**Figure 5:**
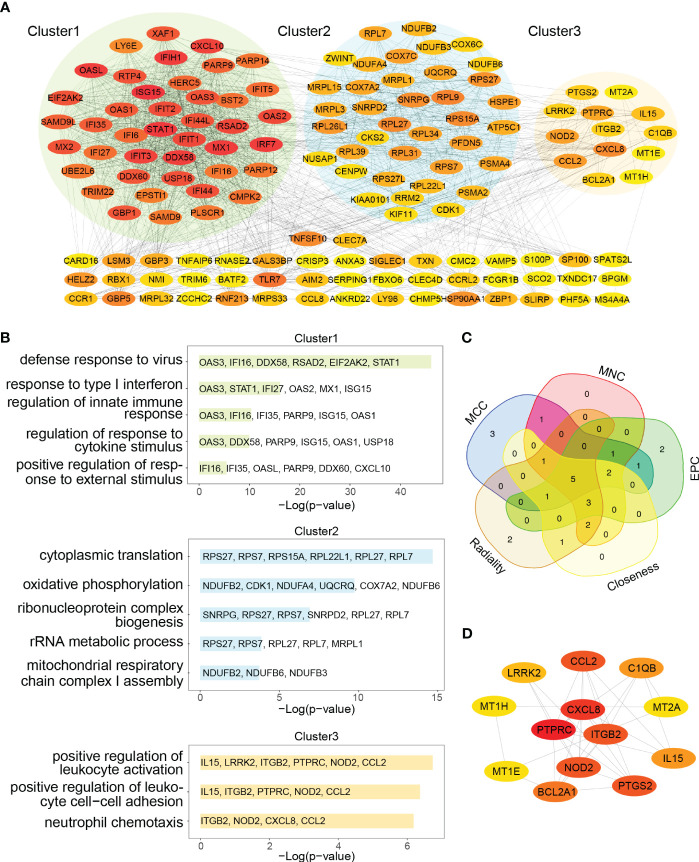
PPI network and Venn diagram of shared genes among SLE and pSS. **(A)** PPI network of combined common module genes and DEGs. The network has 140 nodes and 1610 edges. **(B)** GO analysis of three clusters. **(C)** The Venn diagram showed 5 overlapping genes screened by 5 algorithms. **(D)** PPI network of cluster 3. DEGs, differentially expressed genes; GO, gene ontology; SLE, systemic lupus erythematosus; pSS, primary Sjögren’s syndrome.

The 91 commonly upregulated DEGs and 96 shared genes determined by the positively correlated modules in both autoimmune diseases were combined to yield 152 candidate genes for the subsequent analyses. Subsequently, a PPI network of the candidate genes was constructed, and the three clustering modules from closely connected genes were further extracted through MCODE analysis (**Figure 5A**). Cluster 1 contained 42 nodes and 789 edges. Enrichment analysis results showed that the genes in cluster 1 were mainly associated with type I IFN and cytokine stimulus response. Cluster 2 comprised 38 nodes and 282 edges, and linked to cellular organismal processes. Cluster 3 contained 13 nodes and 33 edges, and involved in leukocyte activation (**Figure 5B**). Thus, clusters 1 and 3 were considered as key modules that may play crucial roles in disease development. To identify the top 15 genes in cluster 1, we utilized five algorithms of the plug-in cytoHubba (MCC, MNC, EPC, Closeness and Radiality) (**Supplementary Table 2**). By intersecting the Venn diagrams, we identified 5 common genes (*IFI44L, ISG15, IFIT1, USP18* and *RSAD2*) in cluster 1 (**Figure 5C**). For cluster 3, we selected the top three genes (*PTPRC, CXCR8* and *ITGB2*) for subsequent analyses (**Figure 5D**).

### Validation of hub genes expression

**Figure 6:**
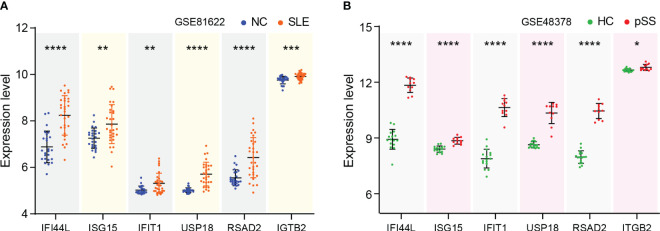
Verification of hub genes expression. **(A)** Expression of hub genes verified in GSE81622. **(B)** Expression of hub genes verified in GSE48378. The comparison in the two sets of data used the mean t-test, separately; *p* -value < 0.05 was considered statistically significant. *p < 0.05, **p < 0.01, ***p < 0.001, ****p < 0.0001.

The expression levels of eight genes were verified in SLE dataset GSE81622 and pSS dataset GSE48378. The results demonstrated that *IFI44L, ISG15, IFIT1, USP18, RSAD2* and *ITGB2* were significantly upregulated in SLE (**Figure 6A**). Additionally, the expression levels of these genes in pSS were also higher than those in healthy control samples (**Figure 6B**). The expression of *PTPRC* and *CXCR8* showed no significant difference in both diseases. Consequently, *IFI44L, ISG15, IFIT1, USP18, RSAD2* and *ITGB2* were identified as hub genes for subsequent analyses (**Supplementary Table 3**). A t-test was conducted to compare the two subsets in these each dataset, separately. A significance level of *p* < 0.05 was applied.

### Pathways involvement and correlation with hub genes

**Figure 7:**
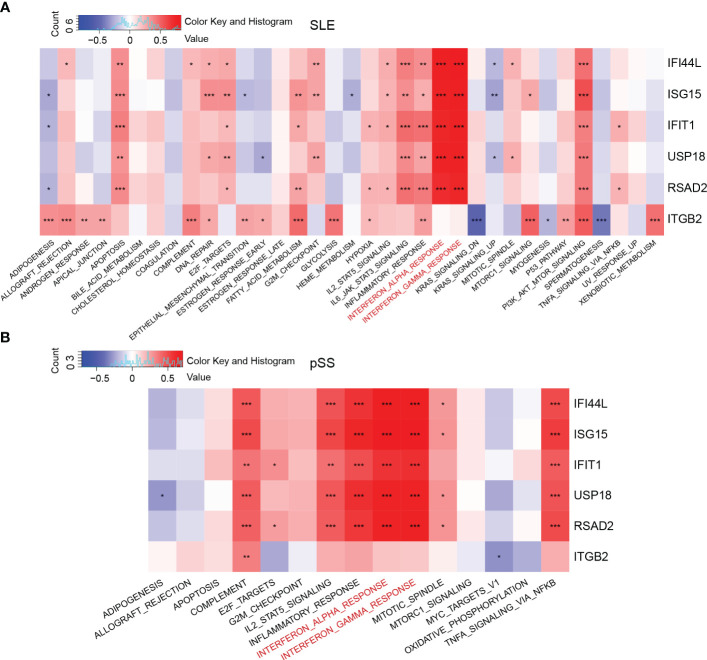
Correlation matrix between hallmark pathways and hub genes. **(A)** Correlation matrix of hallmark pathways and hub genes in SLE. **(B)** Correlation matrix of hallmark pathways and hub genes in pSS. Red: positive correlation; Blue: negative correlation. SLE, systemic lupus erythematosus; pSS, primary Sjögren’s syndrome. *p < 0.05, **p < 0.01, ***p < 0.001.

GSVA was performed to identify the relevant pathways, and Pearson correlation analysis was employed to evaluate the correlation between hub gene and relevant pathway in SLE and pSS (**Figure 7**). A total of 50 hallmark pathways were subjected to GSVA analysis. Overall, the results suggested a strong and consistent correlation between the hub genes (*IFI44L, ISG15, IFIT1, USP18*, and *RSAD2*) and the INTERFERON_ALPHA_RESPONSE, INTERFERON_GAMMA_RESPONSE pathways in both SLE and pSS.

### The expression of hub genes in single-cell RNA-Seq datasets

**Figure 8:**
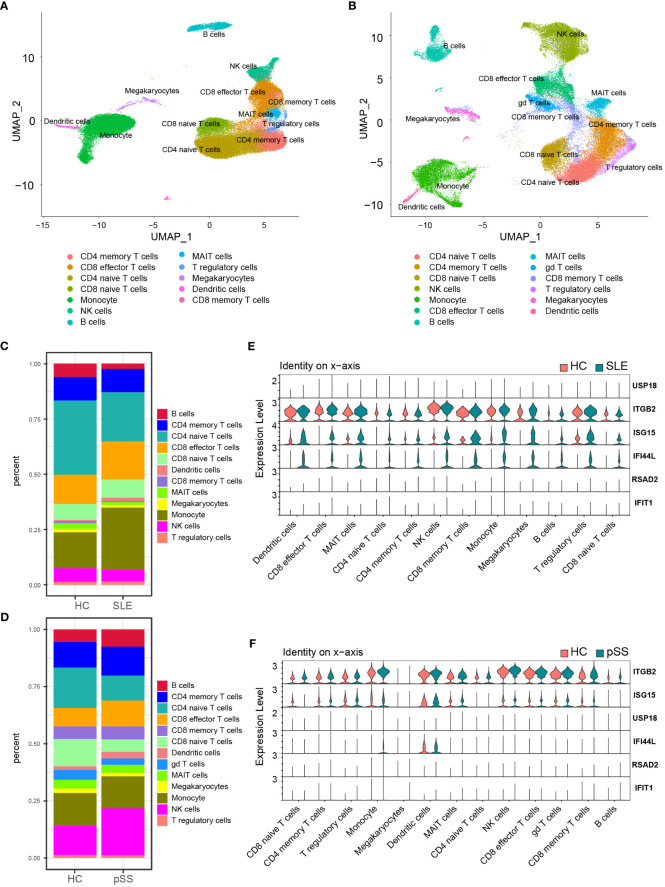
Validation of hub genes in scRNA-seq datasets. **(A)** UMAP visualization GSE157278 scRNA-seq datasets. **(B)** UMAP visualization GSE135779 scRNA-seq datasets; Different colors indicate distinct cell types. **(C)** Cellular composition in SLE and HCs group **(D)** Cellular composition in pSS and HCs group. The colors represent different cell types. **(E)** Violin plot of hub genes expression in different cell types in SLE. **(F)** Violin plot of hub genes expression in different cell types in pSS. SLE, systemic lupus erythematosus; pSS, primary Sjögren’s syndrome.

The PBMC scRNA-seq datasets GSE135779 and GSE157278 were downloaded for subsequent analyses. We selected 5 healthy controls and 7 adults with SLE from the GSE135779 dataset, while the GSE157278 dataset contained 5 pSS patients and 5 normal controls. The two datasets were analyzed separately. Following the Seurat pipeline, and combining the SingleR algorithm with canonical gene markers including *CD3E, CD3D, CD4, CD8A, CCR7, SELL, S100A4, CD79A, MS4A1, GNLY, NCAM1, NKG7, GZMK, GZMB, CD14, LYZ, FCGR3A, MS4A7, FCER1A, CD1C, CLEC4C, LILRA4, PPBP, PF4, SLC4A10, TRDC, TRDV2, FOXP3* as well as *IL2RA*, we identified cell populations. Among the identified populations were CD4 naïve T cells, CD4 memory T cells, CD8 naïve T cells, CD8 memory T cells, CD8 effector T cells, monocytes, NK cells, B cells, DCs and some other cells in the two datasets (**Figures 8A, B**). The dot plot depicted the cell-type-specific markers (**Supplementary Figure 4**). Cell composition analysis revealed that monocytes (HC, 16.3%; SLE, 28.1%) and CD8 effector T cells (HC, 13.3%; SLE, 17.3%) were expanded, while CD4 naïve T cells (HC, 33.5%; SLE, 22.2%.) were decreased in SLE patients compared to HCs (**Figure 8C**). For the pSS dataset, NK cells (HC, 12.9%; pSS, 20.6%), B cells (HC, 5.4%; pSS, 7.5%) and CD8 effector T cells (HC, 8.0%; pSS, 11.3%) were expanded, while CD4 naïve T cells (HC, 17.7%; pSS, 10.9%) and CD8 naïve T cells (HC, 12.0%; pSS, 5.5%) were decreased in pSS patients compared to HCs (**Figure 8D**). The violin plot showed that the expression levels of three hub genes (*IFI44L, ISG15* and *ITGB2*) were elevated in both SLE and pSS in most cell types, especially in monocytes, NK cells and CD8 effector T cells. (**Figures 8E, F**). In summary, the results showed that the proportion of CD8 effector T cells increased, however the proportion of CD4 naïve T cells decreased in SLE and pSS patients. We performed DEG analysis in scRNA-seq datasets. For SLE dataset GSE135779, 230 DEGs were upregulated, meanwhile 537 DEGs were upregulated in pSS dataset GSE157278. After performing an intersection of the upregulated DEGs, 97 shared upregulated DEGs were identified (**Supplementary Figure 5A**). The GO results showed that the shared upregulated DEGs were mainly associated with IFN response (**Supplementary Figure 5B**), which was consistent with the results of WGCNA and DEGs analysis in GSE50772 and GSE84844 datasets. The results of GO analysis on the upregulated DEGs in SLE and pSS were highly consistent with the previous results (**Supplementary Figures 5C, D**). We conducted Hallmark annotation analysis on the upregulated DEGs in all cell types (**Supplementary Figures 5E, F**). The results consistently showed enrichment in the IFN response across all cell types in both SLE and pSS.

### Immune cell fractions and the correlation with hub genes

**Figure 9:**
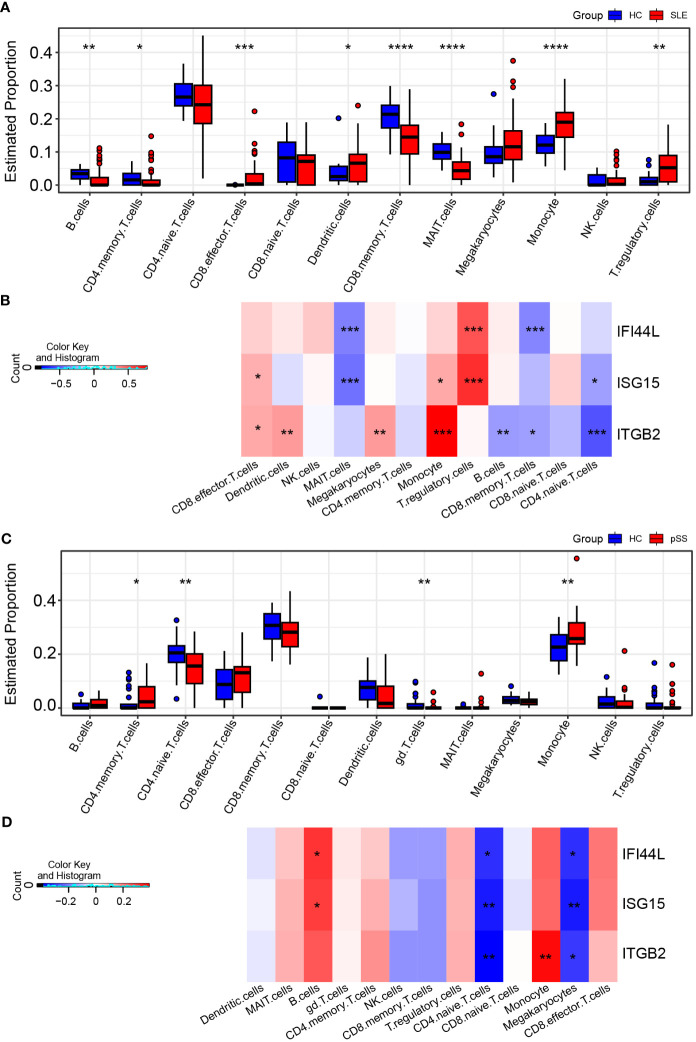
Landscape map of IC in SLE and pSS datasets. **(A)** Boxplot showing the differences of IC between SLE and HC. **(B)** Correlation matrix between IC and hub gene in SLE. **(C)** Boxplot showing the differences of IC between pSS and HC. **(D)** Correlation matrix between IC and hub gene in pSS. Red: positive correlation; blue: negative correlation. SLE, systemic lupus erythematosus; pSS, primary Sjögren’s syndrome. *p < 0.05, **p < 0.01, ***p < 0.001, ****p < 0.0001.

The CIBERSORTx method was used to evaluate the immune cell (IC) composition in peripheral blood using GSE135779 and GSE157278 scRNA-seq datasets as reference matrices for deconvolution on the SLE (GSE50772) and pSS (GSE84844) datasets separately. The boxplot showed that the proportion of CD4 naïve T cells in SLE samples was lower than that in HC samples, despite lack of statistical significance. Interestingly, CD8 effector T cells and monocytes were significantly increased in SLE patients compared to HCs (*p* < 0.05) (**Figure 9A**). Furthermore, Pearson correlation analysis was performed to investigate the correlations between hub genes and ICs in SLE. The heatmap revealed that three hub genes (*IFI44L, ISG15* and *ITGB2*) had positive correlations with monocytes and CD8 effector T cells, while having negative correlations with CD4 naïve T cells (*p* < 0.05) (**Figure 9B**).

In comparison to HCs, the proportion of monocytes exhibited significant increase in pSS samples (*p* < 0.05). Additionally, B cells and CD8 effector T cells displayed increasing trend in pSS, though statistically insignificant. More importantly, CD4 naïve T cells exhibited significant decrease in pSS (**Figure 9C**). The correlations between hub genes and ICs in pSS demonstrated that three hub genes (*IFI44L, ISG15* and *ITGB2*) had positive correlations with monocytes, B cells and CD8 effector T cells, while had significant negative correlations with CD4 naïve T cells (*p* < 0.05) (**Figure 9D**). In summary, the results showed a consistent pattern of increase in CD8 effector T cells, and decrease in CD4 naïve T cells in both SLE and pSS patients, which was consistent with our results of scRNA-seq analysis. Meanwhile, hub genes (*IFI44L, ISG15* and *ITGB2*) exhibited positive correlations with monocytes in SLE and pSS, especially *ITGB2*. The correlations between genes (*IFIT1, USP18* and *RSAD2*) and ICs are provided in **Supplementary Figure 6**.

### Single-cell analysis for the expression of related pathways

**Figure 10:**
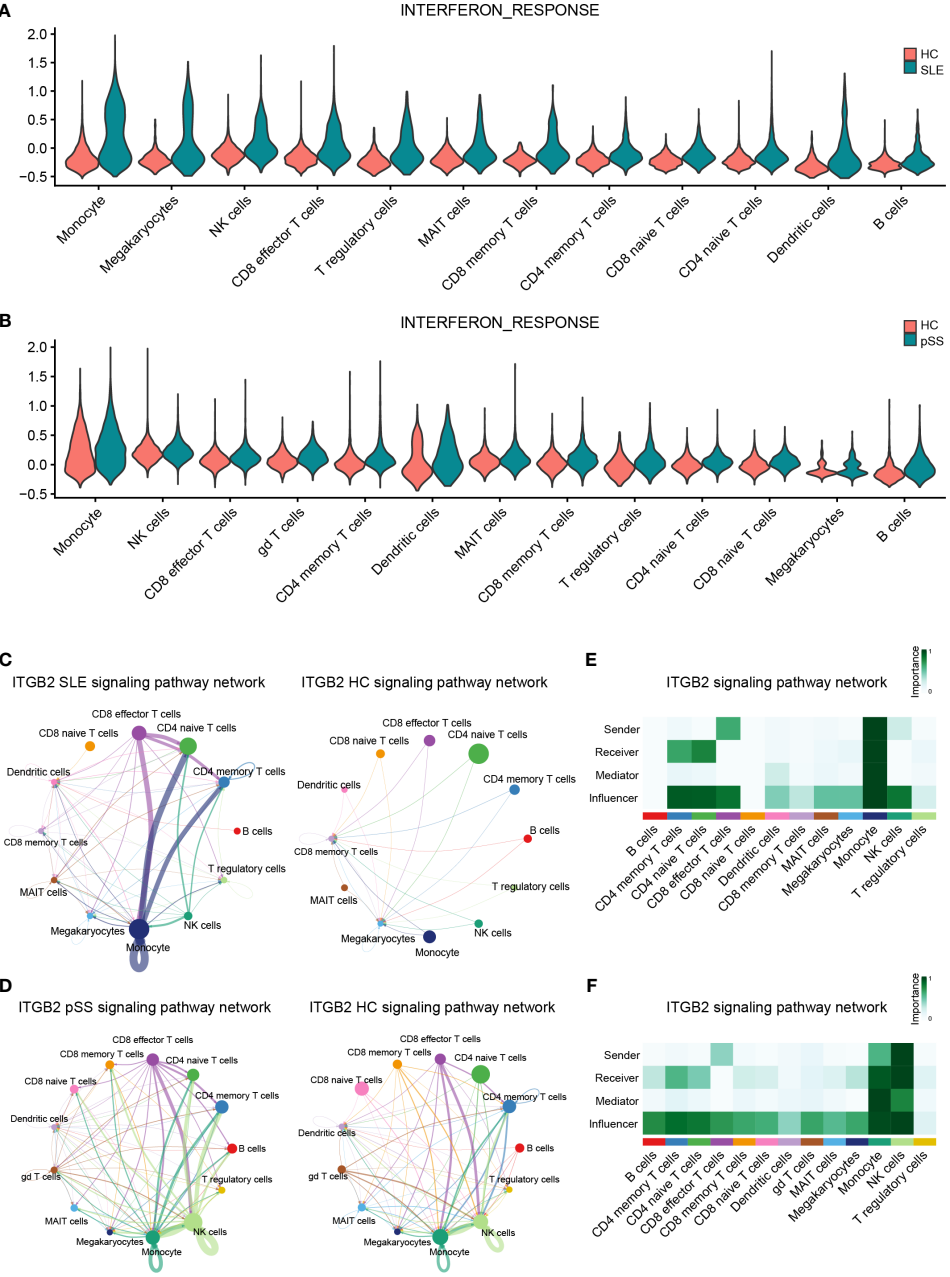
Verification of related pathways in scRNA-seq datasets. **(A)** Violin plot of INTERFERON_RESPONSE expression in SLE. **(B)** Violin plot of INTERFERON_RESPONSE expression in pSS. **(C)** Circos plot showing the ITGB2 signaling pathway network across major cell types in SLE and HCs. **(D)** Circos plot showing the ITGB2 signaling pathway network across major cell types in pSS and HCs. **(E)** Heatmap showing the relative importance of each cell type based on the computed four network centrality measures of the ITGB2 signaling pathway in SLE. **(F)** Heatmap showing the relative importance of each cell type based on the computed four network centrality measures of the ITGB2 signaling pathway in pSS. SLE, systemic lupus erythematosus; pSS, primary Sjögren’s syndrome.

According to the previous GSVA results (**Figures 7A, B**), hub genes (*IFI44L* and *ISG15*) exhibited significant positive correlations with INTERFRON_ALPHA_RESPONSE and INTERFRON_GAMMA_RESPONSE pathways. Therefore, an evaluation of the expression level of INTERFRON RESPONSE in SLE and pSS was performed. We discovered that the INTERFRON RESPONSE was increased in both SLE and pSS patients, particularly in monocytes (**Figures 10A, B**). Besides INTERFRON RESPONSE, we also explored and identified *ITGB2* as a hub gene. Furthermore, we used CellChat to investigate the putative interactions among the major cell types in disease versus control. The results showed that the activity of ITGB2 signaling pathway was increased in SLE and pSS patients, and the ITGB2 signaling pathway was most enriched from monocytes to CD4 T cells and CD8 effector T cells (**Figures 10C, D**). The *ITGB2, ICAM1, ICAM2, CD226* and *ITGAL* expression levels related to ITGB2 signaling pathway were verified both in scRNA-seq and microarray datasets between disease conditions and healthy controls (51, 52) (**Supplementary Figure 7**). The results demonstrated that ITGB2 signaling pathway related genes were upregulated both in SLE and pSS patients, though some were not statistically significantly so. Further analysis showed monocytes are the prominent sender and influencer of the ITGB2 signaling pathway (**Figures 10E, F**). The results indicated that monocytes may play vital roles in IFN response and ITGB2 signaling pathway in the pathogenesis of SLE and pSS, which were consistent with our results of immune cell analysis.

### Prediction and verification of TFs

**Figure 11:**
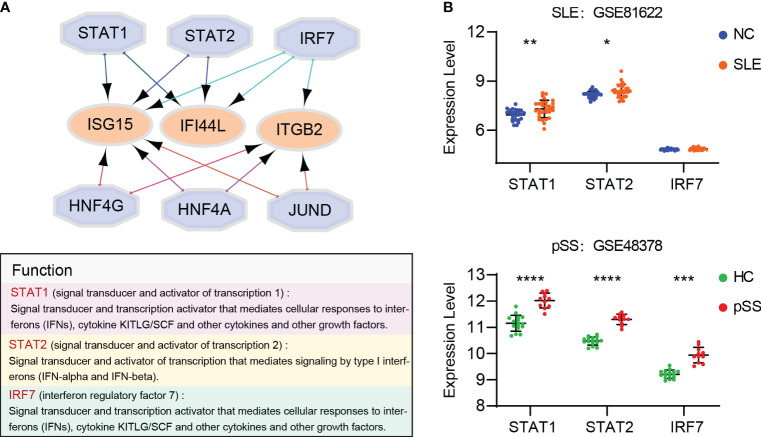
Prediction and verification of TFs. **(A)** iRegulon plug-in predicted TFs of hub genes. **(B)** Expression of TFs verified in GSE81662 and GSE48378. SLE, systemic lupus erythematosus; pSS, primary Sjögren’s syndrome. *p < 0.05, **p < 0.01, ***p < 0.001, ****p < 0.0001.

**Figure 12:**
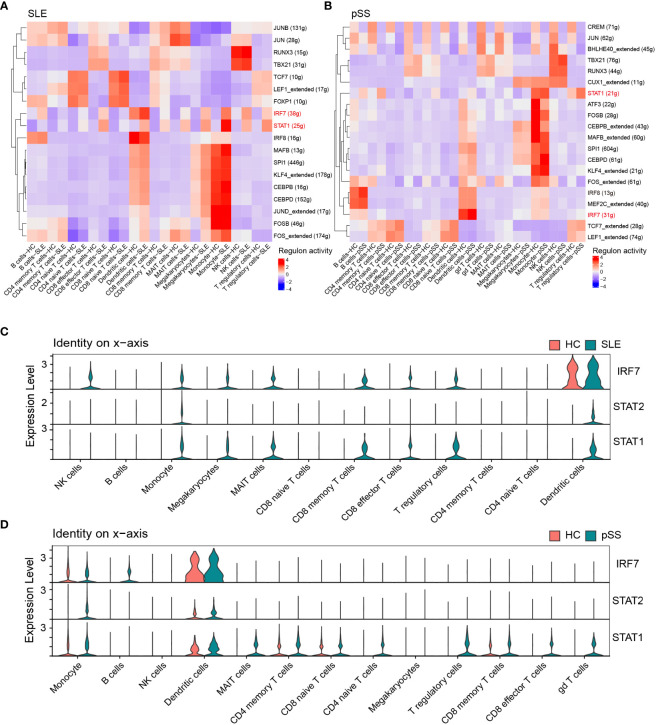
Verification of TFs in scRNA-seq datasets. **(A)** SCENIC analysis revealed TF regulatory information of each cell type in SLE. **(B)** SCENIC analysis revealed TF regulatory information of each cell type in pSS. Red: up-regulated TFs; blue: down-regulated TF. **(C)** Violin plot of TF expression in different cell types in SLE. **(D)** Violin plot of TF expression in different cell types in pSS. SLE, systemic lupus erythematosus; pSS, primary Sjögren’s syndrome.

Based on the iRegulon algorithm, we have identified the top 6 TFs that may regulate the expression of hub genes (*IFI44L, ISG15* and *ITGB2*) (**Figure 11A**). We found that three TFs (STAT1, STAT2 and IRF7) were highly expressed in SLE and pSS validation datasets (**Figure 11B**). To further validate our findings, we employed SCENIC to infer the TF regulatory information underlying each cell type. Remarkably, the SCENIC analysis revealed that STAT1 was upregulated in both diseases and mainly concentrated in monocytes and DCs. Additionally, IRF7 was upregulated and concentrated in DCs in SLE (**Figures 12A, B**). The violin plot showed that expression levels of 3 TFs (STAT1, STAT2 and IRF7) were significantly elevated in SLE and pSS, especially IRF7 in DCs (**Figures 12C, D**).

## Discussion

SLE and pSS are chronic autoimmune diseases predominantly affecting women and exhibit overlapping clinical and serologic characteristics. In a subset of pSS patients, the disease may progress to clinical manifestations, serological profiles and immunological characteristics shared with SLE, leading to fulfillment of classification criteria for both diseases. This condition is commonly referred to as pSS/SLE overlap (53). Despite the increasing knowledge regarding environmental triggers and epigenetic mechanisms, the genetic factors underlying SLE and pSS remain elusive. In this study, we aimed to investigate common target genes, relevant pathways and TFs in SLE and pSS through integrative bioinformatic analyses of transcriptomes. Firstly, we conducted analyses of common genes in the WGCNA module genes and shared DEGs of SLE and pSS. Enrichment analysis showed that these genes were involved in both type I IFN response and cytokine-mediated signaling pathway. Subsequently, we combined the common genes in WGCNA and DEGs, and obtained 152 shared candidate genes. Next, we identified 6 hub genes (*IFI44L, ISG15, IFIT1, USP18, RSAD2* and *ITGB2*) by the PPI network and cytoHuba algorithms, and verified their expression levels. The expression of hub genes was further verified in scRNA-seq datasets. The results showed that 3 hub genes- *IFI44L, ISG15* and *ITGB2*- were upregulated in disease groups. Additionally, we evaluated the correlations between hub genes and ICs as well as related pathways. The results showed that hub genes (*IFI44L* and *ISG15*) had positive correlations with monocytes, as well as the IFN response pathway. *ITGB2* had a significant positive correlation with monocytes and mainly involved in ITGB2 signaling pathway. The IFN response and ITGB2 signaling pathway were increased and enriched in monocytes in SLE and pSS. Finally, TFs (STAT1, STAT2 and IRF7) were predicted and verified, and only STAT1 and IRF7 were upregulated in scRNA-seq data. Notably, IRF7 was specially enriched in DCs.

The biological processes involved in the IFN response, inflammatory, immune response and T cells activation were enriched among the upregulated DEGs in SLE. Activated IFN response has been well recognized as an important feature in SLE (54). The abnormal activation of T cells appears to be involved in the pathogenesis of SLE. An analysis of lymphocyte composition revealed a reduction in naïve CD4 T cells and an increase in CD8 T cells in SLE patients (55). The autoantibodies and immune complex mediated cytokines, such as IL-1, would cause persistent inflammatory response in SLE (56). Besides, the neutrophil extracellular traps and neutrophil to lymphocyte ratio played essential roles in the pathogenesis of SLE (57, 58). Besides IFN response, the upregulated DEGs associated with TNF response, I-kappa B kinase/NF-kappa B signaling were also identified by GO analysis in pSS patients. Serum level of TNF-α has been identified as the most discriminating factor associated with the presence of interstitial lung disease (ILD) in pSS patients (59). B cell-activating factor of the TNF family (BAFF) may contribute to focal lymphocytic infiltration and is an essential cytokine in pSS physiopathology (60). In PBMC from pSS patients, phosphorylated inhibitor of κB (IκB) kinase (IKK) ϵ (IKKϵ), total IKKϵ, pIKKα/β and pNF-κB p65 were significantly increased compared to healthy controls (61). Knockdown of RSAD2 attenuated pSS B cell hyperactivity via suppressing NF-κB signaling (62). Owing to the multitude of influencing factors observed in previous studies as well as our own analyses, comprehensive understanding of the pathogeneses of SLE and pSS remains an ongoing project. The high IFN response plays a critical role both in SLE and pSS.

IFNs are a class of cytokines that exhibit antiviral effects and are induced by viral infections, ultimately leading to the expression of IFN-stimulated genes (ISGs) and further exerting antiviral effects (63, 64). Type I IFNs, including IFN-α, IFN-β, IFN-ϵ and IFN-κ, are the primary interferons capable of exerting antiviral effects. Studies have reported that IFNs can not only act on viruses to interfere with their replication but enhance cellular immunity by acting on T/B cell proliferation and differentiation (65, 66). Type I IFNs stimulate monocytes differentiation and induce immature DCs to express chemokines and costimulatory molecules, which contributes to the pathogenesis of SLE (67). BAFF is stimulated by type I IFNs and promotes B-cell activation, involved in the pathogenesis of pSS (68). Our enrichment analysis of common upregulated DEGs and overlapping genes from positively correlated modules further demonstrates the importance of the type I IFN response in diseases. We also employed GSVA and found hub genes exhibited significant positive correlations with IFNα and IFNγ response pathways. Moreover, a meta-analysis of transcriptomes has identified shared type I IFN- stimulated genes among rheumatoid arthritis (RA), SLE and pSS, such as *IFI44L, IFI44, IFI27* and *IFIT1* (9). Unlike previous studies, our research employed comprehensive and improved bioinformatic methods, and paid more attention to the exploration of hub genes, related pathways and TFs in peripheral blood that are common in SLE and pSS (69, 70). We identified 3 hub genes (*IFI44L, ISG15* and *ITGB2*). *IFI44L* is a type I IFN-stimulated gene, which has benn found to be upregulated in patients with pSS and was markedly increased following with either IFN-α or IFN-β stimulation (71). STAT3 promoted the overexpression of *IFI44L* in monocytes, which contributes to the pathogenesis of SLE. *IFI44L* is expected to become a new therapeutic target for SLE treatment (67, 72). Interferon-stimulated gene 15 (*ISG15*) is a ubiquitin-like protein that is conjugated to intracellular target proteins upon activation by IFN-α and IFN-β (73). The expression level of *ISG15* was higher in saliva and serum from pSS patients than from controls. The expression of ISG15 is relatively high in patients with SLE and correlates with disease activity prior to treatment (74). We identified *IFI44L* and *ISG15* as common hub genes in the two diseases.

However, the pathogeneses of SLE and pSS are exceptionally complicated. Besides IFN response, we also explored and identified *ITGB2* as a hub gene. Integrin subunit β2 (*ITGB2*) encodes integrin β2 protein (CD18) (75). Integrins are heterodimeric transmembrane proteins consisting of alpha and beta subunits. Integrins regulate immune cell trafficking by modulating leukocyte adhesion to blood vessels and facilitating their extravasation into tissues. These proteins play important roles in inflammatory and autoimmune responses (76, 77). Behera and colleagues reported that osteopontin can bind αvβ3 integrin and induce JAK2/STAT3 activation in human breast cancer cells (78). Mastrangeli and colleagues reported the binding properties of deamidated IFN-β to αvβ3 integrin in triple-negative breast cancer (79). The link between interferons and integrins remains for further investigation. Beta2-integrins are leukocyte-specific adhesion molecules that are essential for leukocyte trafficking and immune cell activation. As a result, beta2-integrins may be involved in many autoimmune diseases. *ITGB2* was upregulated in PBMCs from systemic sclerosis (SSc) patients, which may participate in immune cell migration to involved tissues. Splenic B cells from NZB/NZW F1 lupus mice showed ITGB2 activation compared to normal C57Bl/6 mice (75, 80, 81). However, there are no studies reporting its role in pSS, which provides a springboard for future research. Our results are the first to demonstrate increased ITGB2 signaling pathway activity, and upregulated *ITGB2* expression in both SLE and pSS patients. Vedolizumab (targets integrin α4β7), and etrolizumab (anti β7) have been approved by the FDA for the treatment of inflammatory bowel disease (IBD), namely ulcerative colitis (UC) and Crohn’s disease (CD). These drugs have demonstrated efficacy with minimal systemic adverse effects (82, 83). The research about integrins antagonists underscores the central role of these proteins in autoimmune diseases. Additionally, organ-specific delivery of drugs to targeted tissue may further increase the therapeutic potential for anti-integrin agents (84). Lifitegrast, a small-molecule inhibitor that targets integrin αLβ2 has been approved for the topical treatment of dry eye disease (DED). Topical application of lifitegrast provides improvement in inferior corneal staining score and eye dryness (85). Our study revealed that *ITGB2* may be a novel therapeutic target in SLE and pSS. Development of drug delivery strategies will provide greater therapeutic opportunities for targeting integrins.

In addition, we also analyzed TFs and verified their expression levels in microarray and scRNA-seq datasets. We found that 6 TFs may regulate the expression of hub genes. Upon further verification, two TFs (STAT1 and IRF7) are highly expressed in SLE and pSS. Signal transducer and activator of transcription (STAT) families and IFN regulatory factor (IRF) have been demonstrated to play essential roles in regulating type I IFN response (86). All STAT family members primarily function within the Janus kinase-signal transducer and activator of transcription (JAK-STAT) pathway (87). IFNs cause STAT activation and subsequently trigger ISG expression (88, 89). STAT1, STAT2 and IRF9 are capable of amplifying JAK-STAT signaling to reinforce IFN response (90). The JAK-STAT pathway transduces intracellular signals of multiple cytokines, and is critical to the pathogenesis of autoimmune diseases. SLE patients showed substantially higher STAT1 in B cells and plasmablasts (91). STAT1 expression is also increased in labial salivary glands from pSS patients (92). Our study confirmed the essential role of STAT1 in both SLE and pSS. STATs, as JAK substrates, have been investigated as attractive therapeutic targets in autoimmune diseases. However, challenges in the development of STAT inhibitors include issues with bioavailability, *in vivo* efficacy and selectivity (93). Thus, Janus kinase inhibitors (Jakinibs), targeting JAK-STAT pathways, hold promise to block STAT expression. Currently, Jakinibs are most commonly used for RA treatment. In SLE, tofacitinib has been used in phases of clinical trials. Lee and colleagues (94) performed a series of experiments to determine the safety and efficacy of filgotinib for pSS treatment, and suggested that filgotinib has potential for pSS treatment. The mammalian IRF family proteins (IRF1-9) are TFs that play crucial roles in connecting microbial signaling to the responses of IFNs, pro-inflammatory cytokines and innate immune responses (95, 96). IRF3 and IRF7 play pivotal roles in the induction of type I IFN gene transcription (97). IRF7 is a lymphoid TF that is constitutively expressed only in B cells, monocytes and plasmacytoid dendritic cells (pDCs), and is particularly highly expressed in pDCs (98), which was consistent with our study. IRF7 was specifically concentrated in DCs from both SLE and pSS. IRFs can induce the expression of ISGs through a pathway that may depend on or be independent of JAK-STAT signaling (99). IRF7 as transcriptional regulators of type I IFNs and certain single nucleotide polymorphisms (SNPs) in IRF7 to the onset of SLE have been substantiated in previous literature (100). However, the limited studies about IRF7 function regulation in SLE are mainly on murine models. With respect to pSS, IRF7 was upregulated in B cells from patients compared from healthy controls (101). In our study, IRF7 was identified as a pivotal TF in both SLE and pSS. Firstly, the functions of TFs need to be further verified with *in vitro* models. Secondly, STAT1 and IRF7 might act as reporter genes for preliminary screening of drug candidates in SLE and pSS diseases in the future.

There are some limitations in our study. Although we employed comprehensive and improved bioinformatic methods and verified our results in other gene expression profiles, the analysis remains speculative. Further experimental research is needed to confirm the findings in this study, which provides a theoretical basis for future research in the field.

## Conclusions

In summary, we explored and identified the shared hub genes, related pathways and TFs in peripheral blood from SLE and pSS patients for the first time. The hub genes (I*FI44L, ISG15* and *ITGB2*) were identified, and relevant pathways (IFN response and ITGB2 signaling pathway) were found in SLE and pSS. In addition, STAT1 and IRF7 were identified as common TFs, associated with monocytes and DCs. Moreover, IRF7 was predominantly expressed in DCs. This study provides novel insights for further pathogenesis studies of SLE and pSS. In conclusion, a better understanding of the pathogenesis of each disease is of fundamental importance for identifying new therapeutic targets and immunomodulatory agents in future management of SLE and pSS.

## Data availability statement

The datasets presented in this study can be found in online repositories. The names of the repository/repositories and accession number(s) can be found in the article.

## Author contributions

These authors contributed equally: YC, HZ and ZW. YC performed data analyses. HZ was responsible for visualization the figures. YC, HZ, ZW, BG, HA-W, YD, OF, JW and WZ wrote and revised the manuscript draft. YS revised and finalized the manuscript. All authors contributed to the article and approved the submitted version.
