# Corrigendum: Exploring the shared molecular mechanisms between systemic lupus erythematosus and primary Sjögren’s syndrome based on integrated bioinformatics and single-cell RNA-seq analysis

**DOI:** 10.3389/fimmu.2023.1339929

**Published:** 2023-12-08

**Authors:** Yanling Cui, Huina Zhang, Zhen Wang, Bangdong Gong, Hisham Al-Ward, Yaxuan Deng, Orion Fan, Junbang Wang, Wenmin Zhu, Yi Eve Sun

**Affiliations:** ^1^ Stem Cell Translational Research Center, Tongji Hospital, School of Medicine, Tongji University, Shanghai, China; ^2^ Shanghai Institute of Stem Cell Research and Clinical Translation, Shanghai East Hospital, School of Medicine, Tongji University, Shanghai, China; ^3^ Division of Rheumatology, Tongji Hospital of Tongji University School of Medicine, Shanghai, China

**Keywords:** systemic lupus erythematosus, primary Sjögren’s syndrome, bioinformatics, hub genes, TFs, scRNA-seq

In the published article, there was an error in **Figures 8C**, **9A, 9B**, **10C, 10E** and **Supplementary Figures S5E**, **S6A**, **S7E** as published. We noticed that a cell type was incorrectly described. The “gd T cells” should be “CD8 memory T cells” in our article since the R codes were not revised in time. The corrected **Figures 8**, **9**, **10** and **Supplementary Figures 5**, **6** and **7** and their captions “FIGURE 8 Validation of hub genes in scRNA-seq datasets”, “FIGURE 9 Landscape map of IC in SLE and pSS datasets”, “FIGURE 10 Verification of related pathways in scRNA-seq datasets”, “Supplementary Figure 5 Functional analysis of upregulated DEGs in scRNA-seq”, “Supplementary Figure 6 Heatmap of correlation matrix”, “Supplementary Figure 7 The expression levels of ITGB2 signaling pathway related genes (ITGB2, ICAM1, ICAM2, CD226 and ITGAL)” appear below or can be found in the Supplementary Material of the original article.

**Figure 8:**
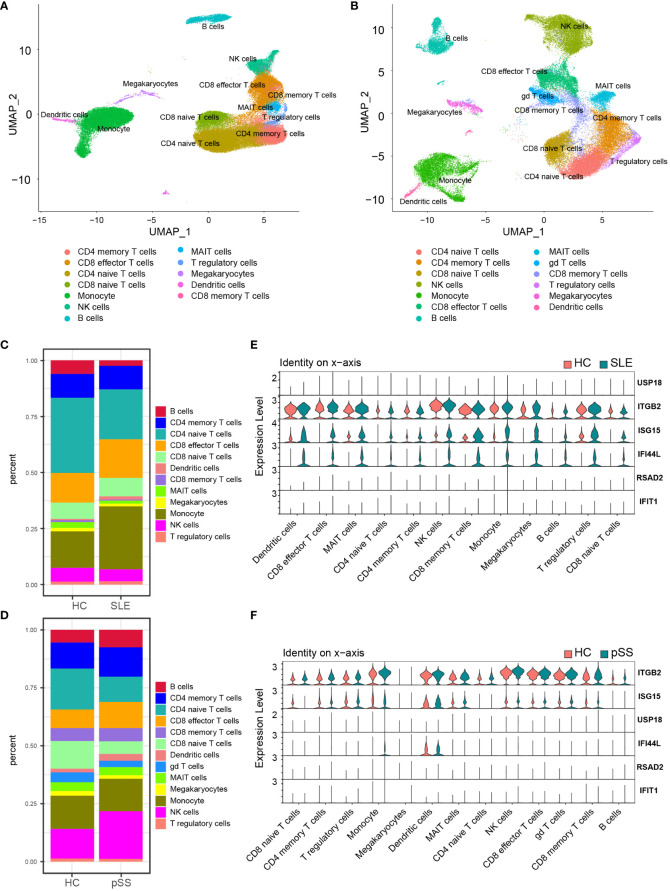
Validation of hub genes in scRNA-seq datasets. **(A)** UMAP visualization GSE157278 scRNA-seq datasets. **(B)** UMAP visualization GSE135779 scRNAseq datasets; Different colors indicate distinct cell types. **(C)** Cellular composition in SLE and HCs group **(D)** Cellular composition in pSS and HCs group. The colors represent different cell types. **(E)** Violin plot of hub genes expression in different cell types in SLE. **(F)** Violin plot of hub genes expression in different cell types in pSS. SLE, systemic lupus erythematosus; pSS, primary Sjögren’s syndrome.

**Figure 9:**
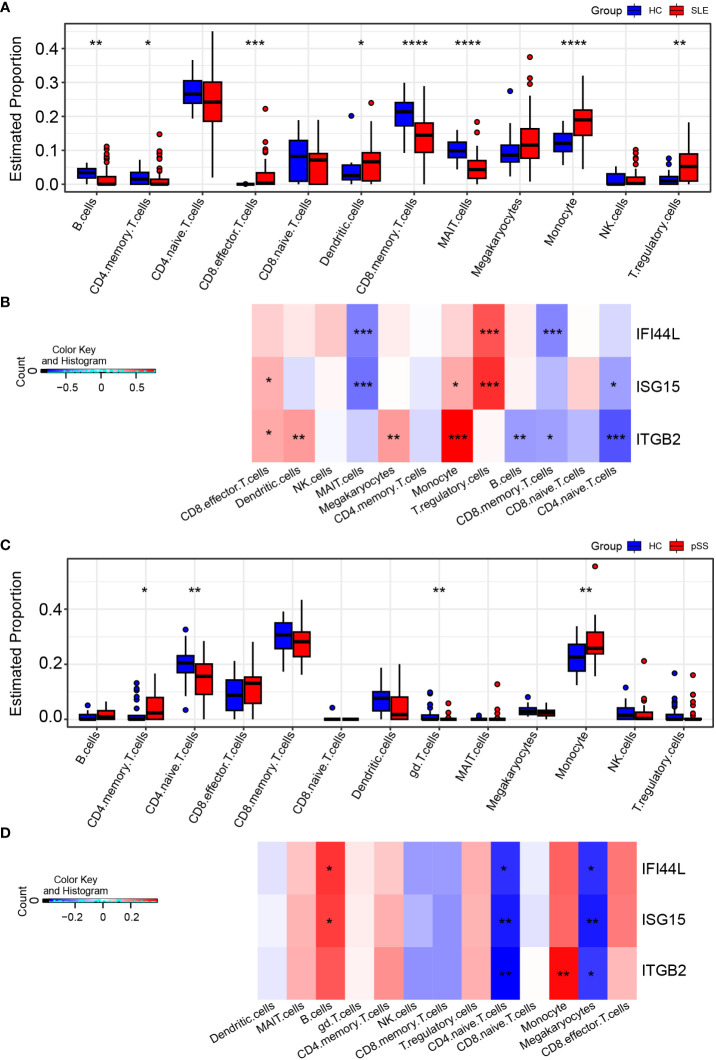
Landscape map of IC in SLE and pSS datasets. **(A)** Boxplot showing the differences of IC between SLE and HC. **(B)** Correlation matrix between IC and hub gene in SLE. **(C)** Boxplot showing the differences of IC between pSS and HC. **(D)** Correlation matrix between IC and hub gene in pSS. Red: positive correlation; blue: negative correlation. SLE, systemic lupus erythematosus; pSS, primary Sjögren’s syndrome. *p < 0.05, **p < 0.01, ***p < 0.001, ****p < 0.0001.

**Figure 10:**
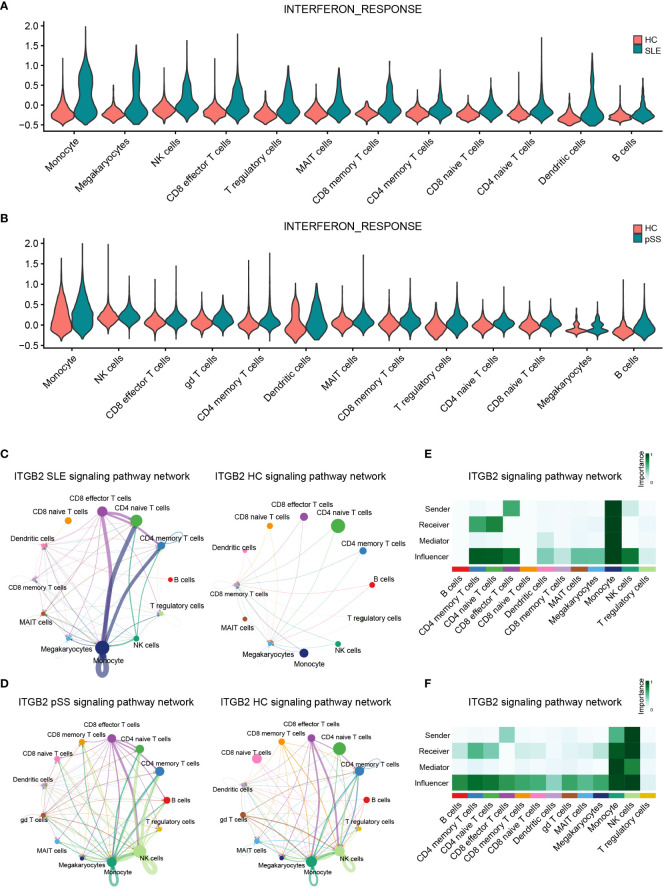
Verification of related pathways in scRNA-seq datasets. **(A)** Violin plot of INTERFERON_RESPONSE expression in SLE. **(B)** Violin plot of INTERFERON_RESPONSE expression in pSS. **(C)** Circos plot showing the ITGB2 signaling pathway network across major cell types in SLE and HCs. **(D)** Circos plot showing the ITGB2 signaling pathway network across major cell types in pSS and HCs. **(E)** Heatmap showing the relative importance of each cell type based on the computed four network centrality measures of the ITGB2 signaling pathway in SLE. **(F)** Heatmap showing the relative importance of each cell type based on the computed four network centrality measures of the ITGB2 signaling pathway in pSS. SLE, systemic lupus erythematosus; pSS, primary Sjögren’s syndrome.

The authors apologize for this error and state that this does not change the scientific conclusions of the article in any way. The original article has been updated.

