# Supplementary material for: Exploring the shared molecular mechanisms between systemic lupus erythematosus and primary Sjögren’s syndrome based on integrated bioinformatics and single-cell RNA-seq analysis
Source: Front Immunol. 2023 Aug 8;14:1212330. doi: 10.3389/fimmu.2023.1212330 (PMC10442653; doi:10.3389/fimmu.2023.1212330)
Supplement: Supplementary file 4 [file DataSheet_4.docx]

Supplementary Material

Exploring the Shared Molecular Mechanisms Between Systemic Lupus Erythematosus and Primary Sjögren's Syndrome Based on Integrated Bioinformatics and Single-Cell RNA-Seq Analysis

Yanling Cui ^1,2†^, Huina Zhang ^1,2†^, Bangdong Gong^3^, Hisham Al-Ward ^1,2^, Yaxuan Deng ^1,2^, Junbang Wang ^1^, Yi Eve Sun ^1,2*^

*** Correspondence:** Yi Eve Sun*****: [yi.eve.sun@gmail.com](mailto:yi.eve.sun@gmail.com)

**Supplementary Figure 1:** Clustering of samples.


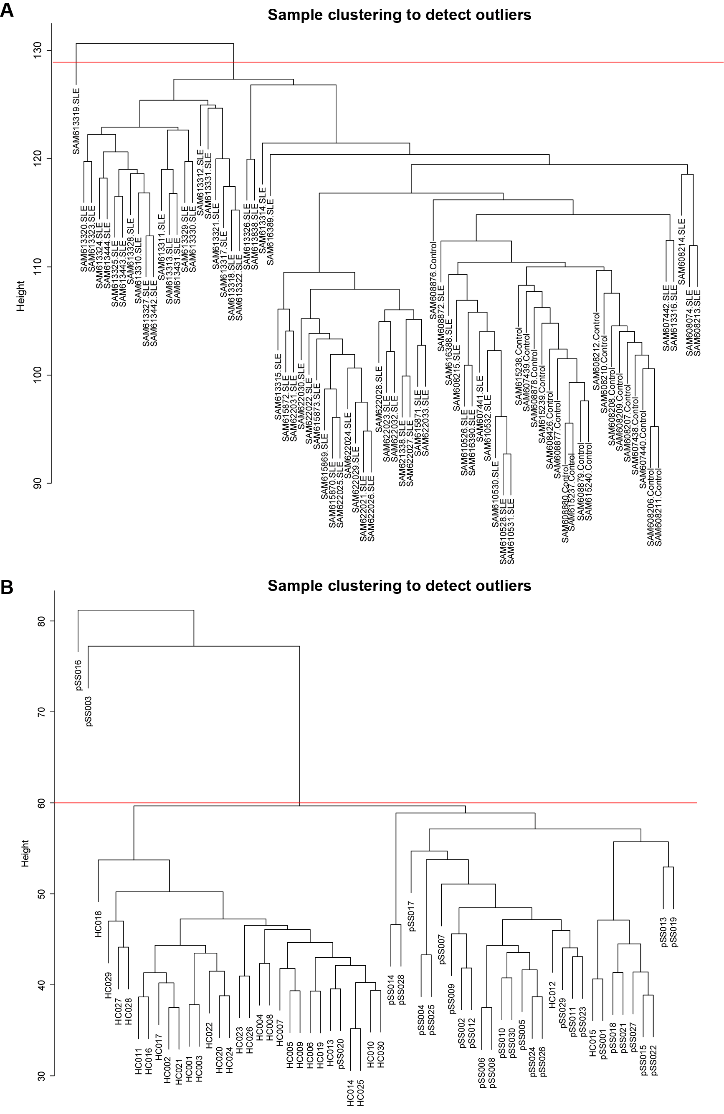


**(A)** Clustering of SLE samples according to the gene expression level in GSE50772. **(B)** Clustering of pSS samples according to the gene expression level in GSE84844. Each branch represents a sample in the datasets, and red line represent the value of cut Height. SLE, systemic lupus erythematosus; pSS, primary Sjögren’s syndrome.

**Supplementary Figure 2:** Venn diagram and GO analysis.


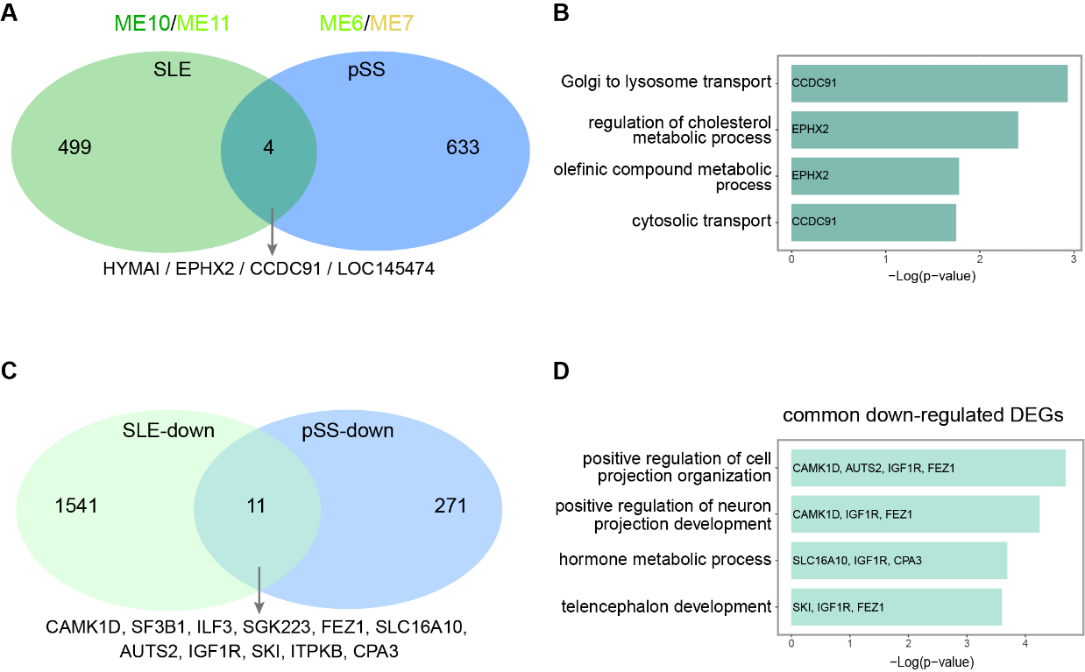


**(A)** Venn diagram showing the overlapped gene in negatively correlated modules in SLE and pSS. **(B)** GO analysis of the 4 common genes. Venn diagram **(C)** and GO analysis **(D)** of 11 downregulated DEGs overlapped in SLE and pSS datasets. DEGs, differentially expressed genes; GO, gene ontology; SLE, systemic lupus erythematosus; pSS, primary Sjögren’s syndrome.

**Supplementary Figure 3:** GO analysis of upregulated DEGs.


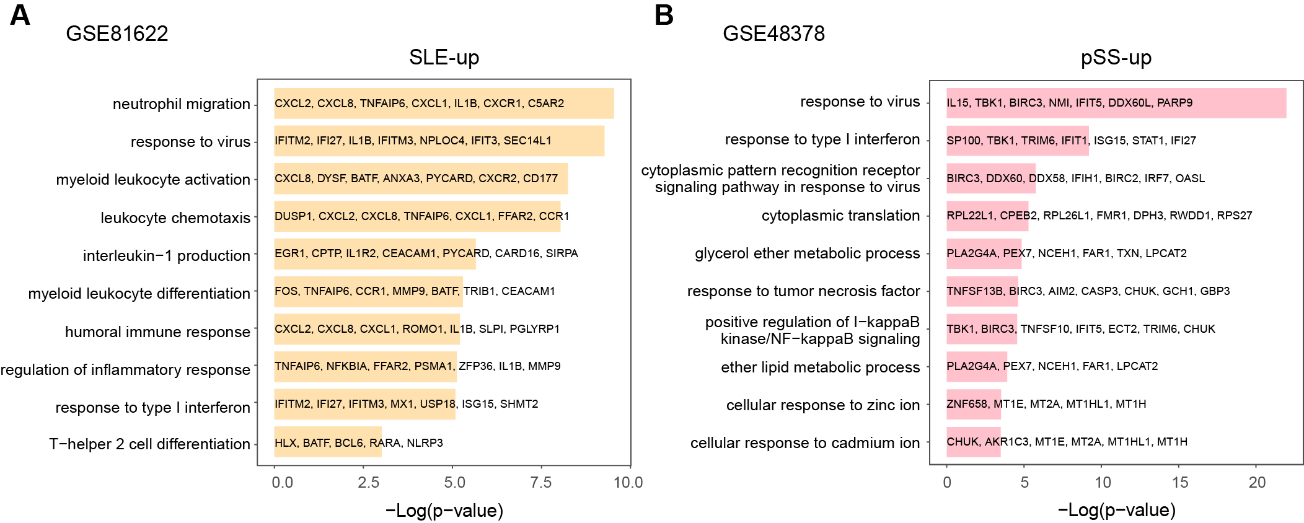


GO analysis of upregulated DEGs in SLE **(A)** and pSS **(B)**. DEGs, differentially expressed genes; GO, gene ontology; SLE, systemic lupus erythematosus; pSS, primary Sjögren’s syndrome.

**Supplementary Figure 4:** Dot plot of cell-type-specific markers.


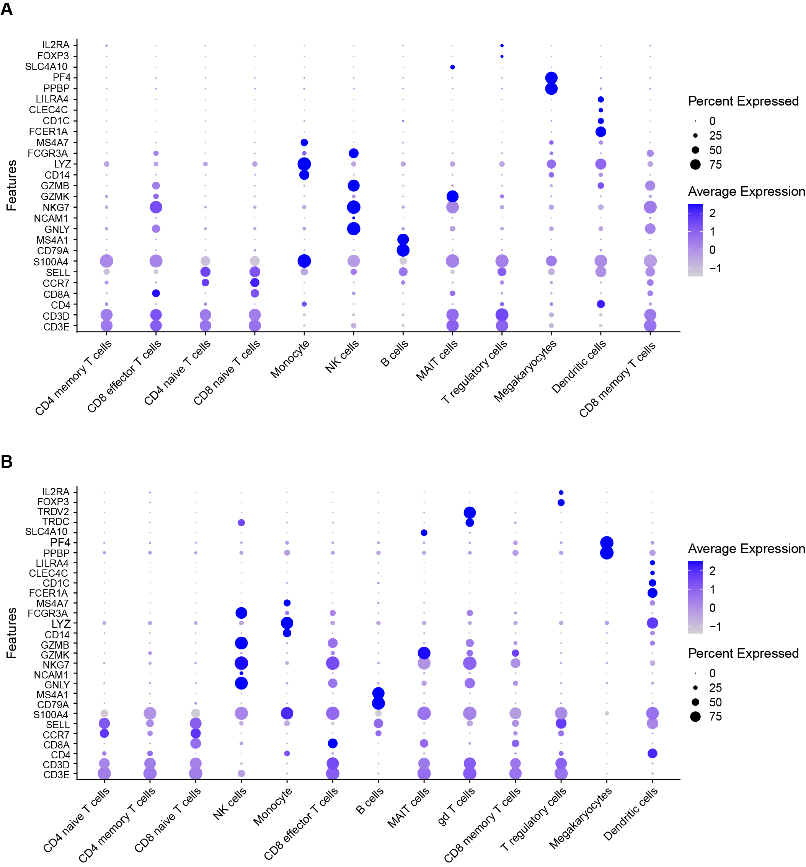


Dot plot of cell-type-specific markers in SLE **(A)** and pSS **(B)**. SLE, systemic lupus erythematosus; pSS, primary Sjögren’s syndrome.

**Supplementary Figure 5:** Functional analysis of upregulated DEGs in scRNA-seq.


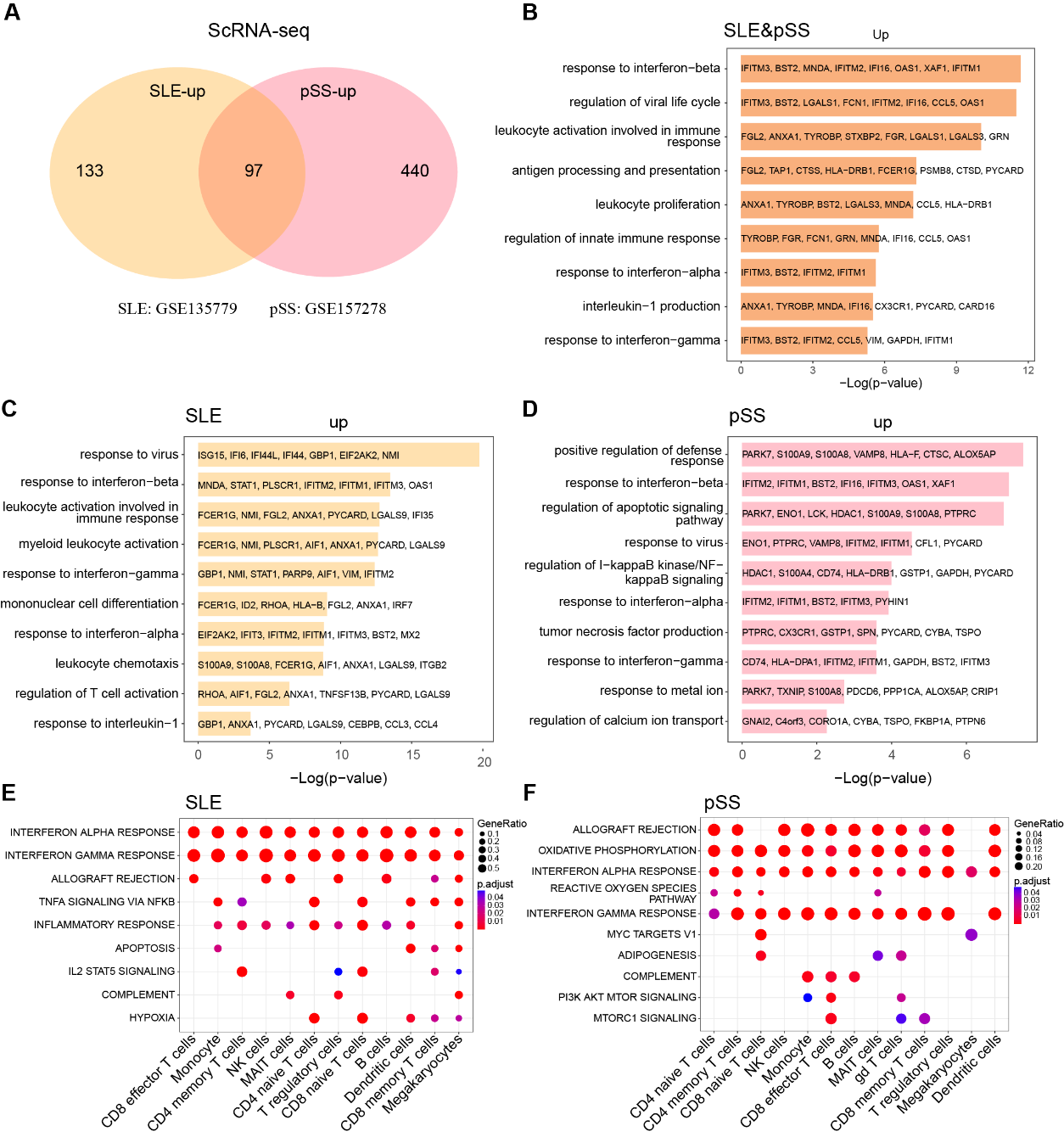


**(A)** 97 upregulated DEGs overlapped in the two datasets. **(B)** GO analysis of common upregulated DEGs. GO analysis of upregulated DEGs in total immune cells in SLE **(C)** and pSS **(D)**. Hallmark annotation of upregulated DEGs in all cell types in SLE **(E)** and pSS **(F)**. DEGs: differentially expressed genes; GO, gene ontology; SLE, systemic lupus erythematosus; pSS, primary Sjögren’s syndrome.

**Supplementary Figure 6:** Heatmap of correlation matrix.


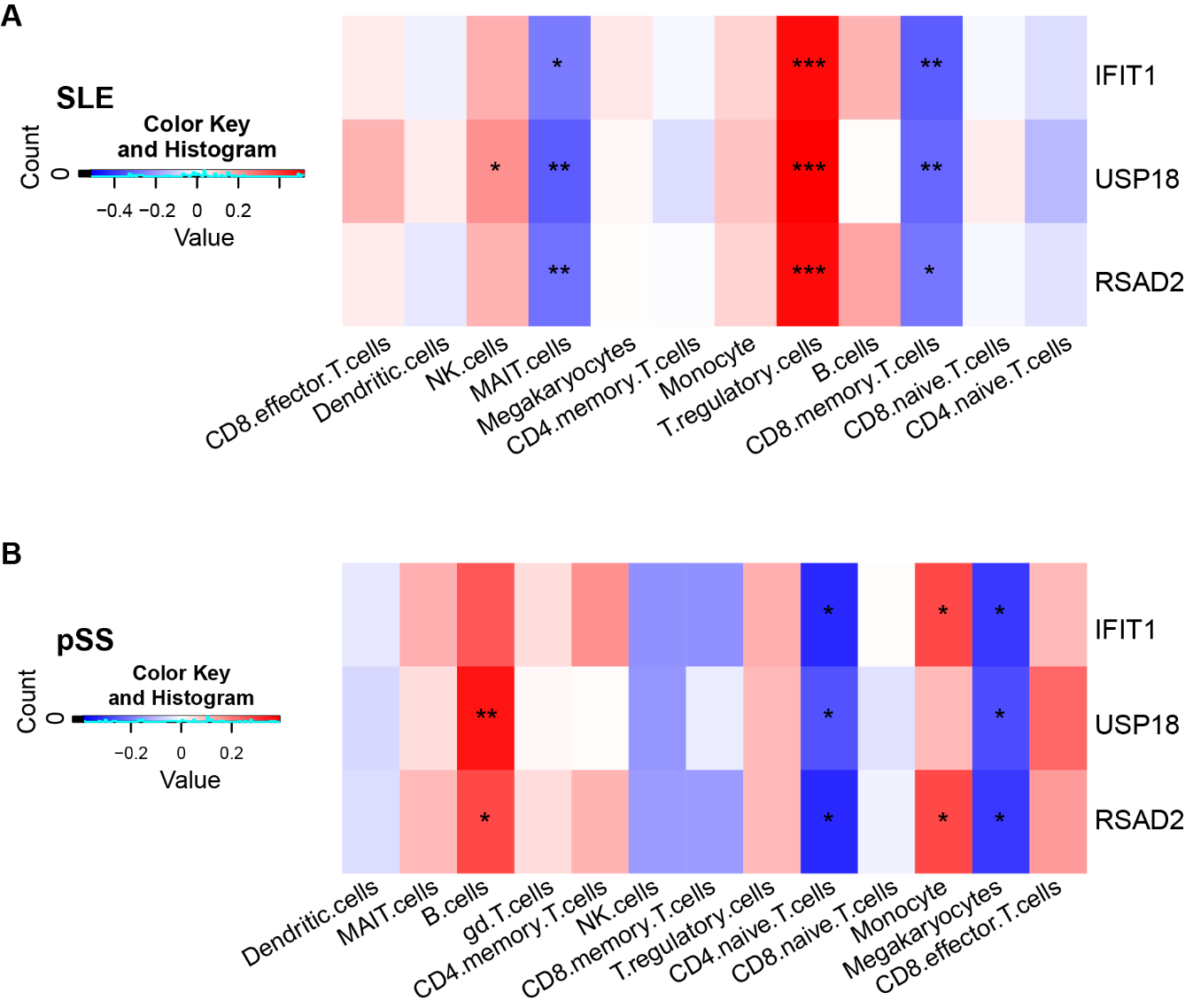


Correlation matrix between IC and genes (*IFIT1, USP18* and *RSAD2*) in SLE **(A)** and pSS **(B)**. Red: positive correlation; blue: negative correlation. SLE, systemic lupus erythematosus; pSS, primary Sjögren’s syndrome. **p* < 0.05, ***p* < 0.01, ****p* < 0.001.

**Supplementary Figure 7:** The expression levels of ITGB2 signaling pathway related genes (*ITGB2, ICAM1, ICAM2, CD226* and *ITGAL*).


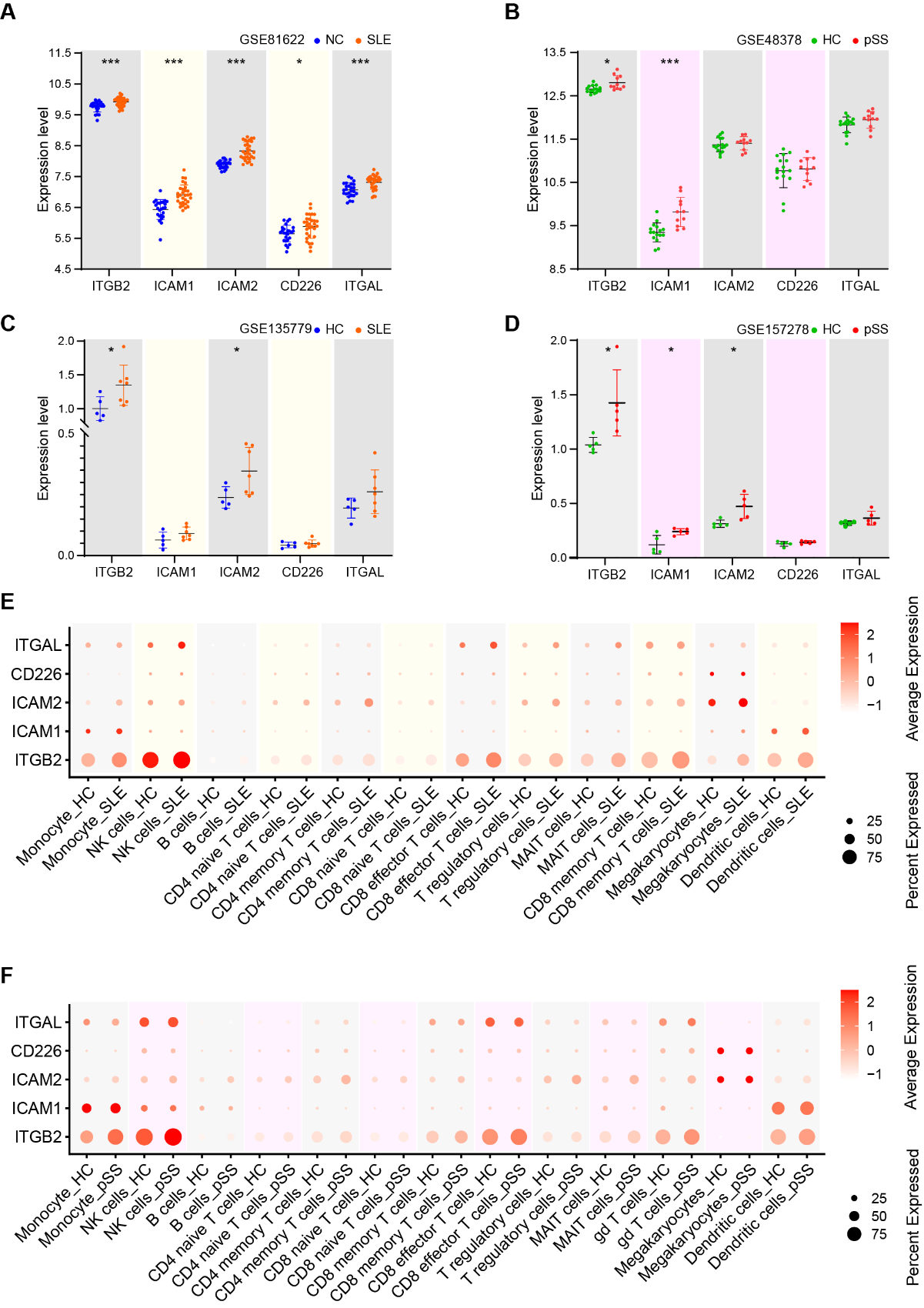


Expression of ITGB2 signaling pathway related genes (*ITGB2, ICAM1, ICAM2, CD226* and *ITGAL*) in GSE81622 **(A)**, GSE48378 **(B)**, GSE135779 **(C)**, and GSE157278 **(D)**. The dot plot of ITGB2 signaling pathway related genes in SLE **(E)** and pSS **(F)**. The comparison in the two sets of data used the mean t-test, separately; SLE, systemic lupus erythematosus; pSS, primary Sjögren’s syndrome. *p* -value < 0.05 was considered statistically signiﬁcant. **p* < 0.05, ***p* < 0.01, ****p* < 0.001, *****p* < 0.0001.
