# Supplementary material for: Exploring the shared molecular mechanisms between systemic lupus erythematosus and primary Sjögren’s syndrome based on integrated bioinformatics and single-cell RNA-seq analysis
Source: Front Immunol. 2023 Aug 8;14:1212330. doi: 10.3389/fimmu.2023.1212330 (PMC10442653; doi:10.3389/fimmu.2023.1212330)
Supplement: Supplementary file 5 [file Table_1.docx]

Supplementary Material

Exploring the Shared Molecular Mechanisms Between Systemic Lupus Erythematosus and Primary Sjögren's Syndrome Based on Integrated Bioinformatics and Single-Cell RNA-seq Analysis

Yanling Cui ^1,2†^, Huina Zhang ^1,2†^, Bangdong Gong^3^, Hisham Al-Ward ^1,2^, Yaxuan Deng ^1,2^, Junbang Wang ^1^, Yi Eve Sun ^1,2*^

*** Correspondence:** Yi Eve Sun*****: yi.eve.sun@gmail.com

**Supplementary Table 1:** Summary of GEO datasets involving SLE and pSS patients.

| No. | GSE number | Platform | Samples | Source types | Disease |
| --- | --- | --- | --- | --- | --- |
| 1 | GSE50772 | GPL570 | 61 patients and 20 controls | PBMCs | SLE |
| 2 | GSE81622 | GPL10558 | 30 patients and 25 controls | PBMCs | SLE |
| 3 | GSE135779 | GPL20301 | 42 patients and 17 controls | PBMCs | SLE |
| 4 | GSE84844 | GPL570 | 30 patients and 30 controls | Whole blood | pSS |
| 5 | GSE48378 | GPL5175 | 11 patients and 16 controls | PBMCs | pSS |
| 6 | GSE157278 | GPL24676 | 5 patients and 5 controls | PBMCs | pSS |

PBMCs: peripheral blood mononuclear cells; SLE, systemic lupus erythematosus; pSS, primary Sjögren’s syndrome.
