# Supplementary material for: Exploring the shared molecular mechanisms between systemic lupus erythematosus and primary Sjögren’s syndrome based on integrated bioinformatics and single-cell RNA-seq analysis
Source: Front Immunol. 2023 Aug 8;14:1212330. doi: 10.3389/fimmu.2023.1212330 (PMC10442653; doi:10.3389/fimmu.2023.1212330)
Supplement: Supplementary file 6 [file Table_2.docx]

Supplementary Material

Exploring the Shared Molecular Mechanisms Between Systemic Lupus Erythematosus and Primary Sjögren's Syndrome Based on Integrated Bioinformatics and Single-Cell RNA-seq Analysis

Yanling Cui ^1,2†^, Huina Zhang ^1,2†^, Bangdong Gong^3^, Hisham Al-Ward ^1,2^, Yaxuan Deng ^1,2^, Junbang Wang ^1^, Yi Eve Sun ^1,2*^

*** Correspondence:** Yi Eve Sun*****: yi.eve.sun@gmail.com

**Supplementary Table 2:** The top 15 hub genes rank in cytoHubba.

| No. | MCC | MNC | EPC | Closeness | Radiality |
| --- | --- | --- | --- | --- | --- |
| 1 | USP18 | CXCL10 | IRF7 | ISG15 | ISG15 |
| 2 | OAS3 | MX1 | USP18 | CXCL10 | CXCL10 |
| 3 | RTP4 | ISG15 | MX1 | MX1 | CXCL8 |
| 4 | OASL | IFIH1 | IFIT1 | IFIT1 | MX1 |
| 5 | IFIT3 | IFIT1 | IFI44L | RSAD2 | IFIT1 |
| 6 | IFIT1 | IRF7 | ISG15 | DDX58 | RSAD2 |
| 7 | OAS2 | USP18 | IFIT3 | IRF7 | DDX58 |
| 8 | RSAD2 | DDX58 | RSAD2 | IFIH1 | IRF7 |
| 9 | ISG15 | RSAD2 | PARP9 | USP18 | IFIH1 |
| 10 | HERC5 | OAS3 | IFIT5 | CXCL8 | CCL2 |
| 11 | IFI44 | RTP4 | IFIH1 | OAS1 | OAS1 |
| 12 | IFI44L | IFI44 | OAS3 | IFI44 | USP18 |
| 13 | XAF1 | IFI44L | XAF1 | IFI44L | IFNG |
| 14 | IFIT2 | IFIT3 | RTP4 | IFIT3 | IFI44 |
| 15 | OAS1 | OAS2 | OAS1 | OAS3 | IFI44L |

MCC: maximal clique centrality; MNC: maximum neighborhood component; EPC: edge percolated component.
