# Supplementary material for: Exploring the shared molecular mechanisms between systemic lupus erythematosus and primary Sjögren’s syndrome based on integrated bioinformatics and single-cell RNA-seq analysis
Source: Front Immunol. 2023 Aug 8;14:1212330. doi: 10.3389/fimmu.2023.1212330 (PMC10442653; doi:10.3389/fimmu.2023.1212330)
Supplement: Supplementary file 7 [file Table_3.docx]

Supplementary Material

Exploring the Shared Molecular Mechanisms Between Systemic Lupus Erythematosus and Primary Sjögren's Syndrome Based on Integrated Bioinformatics and Single-Cell RNA-seq Analysis

Yanling Cui ^1,2†^, Huina Zhang ^1,2†^, Bangdong Gong^3^, Hisham Al-Ward ^1,2^, Yaxuan Deng ^1,2^, Junbang Wang ^1^, Yi Eve Sun ^1,2*^

*** Correspondence:** Yi Eve Sun*****: yi.eve.sun@gmail.com

**Supplementary Table 3:** The details of the hub genes.

| No. | Gene symbol | Full name | Function |
| --- | --- | --- | --- |
| 1 | IFI44L | Interferon Induced Protein 44 Like | Predicted to enable GTP binding activity. Involved in defense response to virus. |
| 2 | ISG15 | ISG15 Ubiquitin Like Modifier | Involved in chemotactic activity towards neutrophils, and antiviral activity during viral infections. |
| 3 | IFIT1 | Interferon-Induced Protein with Tetratricopeptide Repeats 1 | IFIT1 is a interferon-stimulated genes, and encoded protein may inhibit viral replication and translational initiation. |
| 4 | USP18 | Ubiquitin Specific Peptidase 18 | Encodes protein, belongs to the ubiquitin-specific proteases (UBP) family of enzymes that cleave ubiquitin from ubiquitinated protein substrates. This protein efficiently cleaves only ISG15 (a ubiquitin-like protein) fusions, is a major ISG15-specific protease. |
| 5 | RSAD2 | Radical S-Adenosyl Methionine Domain Containing 2 | Involved in cellular antiviral response and innate immune signaling. Inhibits both DNA and RNA viruses, including influenza virus, human immunodeficiency virus (HIV-1) and Zika virus. |
| 6 | ITGB2 | Integrin Subunit Beta 2 | The encoded protein plays an important role in immune response and defects in this gene cause leukocyte adhesion deficiency. ITGB2 is a receptor for ICAM1, ICAM2, ICAM3 and ICAM4, and also a receptor for the secreted form of ubiquitin-like protein ISG15. |
